# Diagnosis-based pathways for conservative and minimally invasive management of temporomandibular disorders: a scoping review

**DOI:** 10.22514/jofph.2026.034

**Published:** 2026-05-12

**Authors:** Mohammad Gazali, Alfan Maulana Erdiansyah, Husni Mubarak, Abul Fauzi, Andi Tajrin, Hasanuddin Hasanuddin, Muhammad Ruslin

**Affiliations:** ^1^Department of Oral and Maxillofacial Surgery, Faculty of Dentistry, Hasanuddin University, 90245 Makassar, Indonesia; ^2^Department of Oral and Maxillofacial Surgery, Hasanuddin University Dental Hospital, 90156 Makassar, Indonesia; ^3^Department of Dental Public Health and Preventive Dentistry, Faculty of Dentistry, Hasanuddin University, 90245 Makassar, Indonesia

**Keywords:** Conservative therapy, DC/TMD, Diagnostic criteria, Minimally invasive treatment, RDC/TMD, Temporomandibular disorders

## Abstract

Temporomandibular disorders (TMDs) encompass a spectrum of musculoskeletal and joint conditions of the masticatory system that frequently lead to chronic pain, limited function, and reduced quality of life. Although conservative and minimally invasive approaches are recommended as first-line therapies, their comparative efficacy remains unclear.This scoping review mapped evidence from randomized controlled trials (RCTs) published between 2015 and 2025 to identify diagnosis-based effective management pathways for TMDs. A comprehensive literature search was conducted using the Scopus, Embase, Web of Science, and PubMed databases for RCTs published within the last 10 years. Eligible studies included adult patients diagnosed with Research Diagnostic Criteria for Temporomandibular Disorders (RDC/TMD) or Diagnostic Criteria for Temporomandibular Disorders (DC/TMD) categories and compared conservative or minimally invasive treatments reporting pain, function, or psychosocial outcomes. Data were extracted and charted according to the Preferred Reporting Items for Systematic Reviews and Meta-Analyses Extension for Scoping Reviews (PRISMA-ScR) guidelines, and findings were organized into diagnosis-based intervention pathways reflecting first-line, adjunctive, and escalation therapies. A total of 129 RCTs met the inclusion criteria, revealing a stepwise treatment pathway across the diagnostic subtypes. Education, behavioral therapy, and exercise are the first-line management options that improve pain intensity and mandibular function. Splint therapy and other physical modalities can enhance pain modulation. Minimally invasive interventions for refractory cases provide various long-term benefits. Visual Analog Scale (VAS) and Maximum Mouth Opening (MMO) improvements were the primary outcomes, indicating pain relief and functional recovery as therapeutic endpoints. Overall, the evidence from the past decade supports a diagnosis-driven, stepwise model that emphasizes conservative multimodal care as the cornerstone of TMD management. Minimally invasive procedures should be reserved for persistent pain after optimized non-surgical therapy. Future RCTs should standardize diagnostic and outcome measures to strengthen evidence-based clinical pathways.

## 1. Introduction

Temporomandibular disorders (TMDs) are complex musculoskeletal and neuromuscular conditions that affect the temporomandibular joints, masticatory muscles, and surrounding structures, frequently resulting in pain, limited mobility, and joint noise [1]. Epidemiological data indicate a significant global burden; a recent meta-analysis estimated the overall prevalence of TMDs to be 34% in the general population, with regional variability [2]. Another worldwide study found that the prevalence was almost 29.5% and that women are more likely to be affected than men [3]. In younger demographics, increasing incidences of painful TMD and their correlation with primary headaches have been reported [4]. The ongoing clinical variability in TMD diagnosis and treatment underscores the need for a systematic framework to address existing issues.

Recent advancements have sought to tackle these challenges through various strategies, particularly by highlighting the biopsychosocial model of TMD, which integrates a holistic, multidisciplinary framework for comprehending the condition [5]. The DC/TMD is a standardized diagnostic framework that includes physical (Axis I) and psychosocial (Axis II) aspects to improve the reliability and validity of the RDC/TMD criteria. This framework is accurate and sensitive for common pain-related TMD diagnoses and supports the subtyping of disorders [1]. Psychological interventions, such as cognitive-behavioral therapy, are gaining popularity for their effectiveness when used in conjunction with biomedical treatments, indicating that a holistic approach can produce substantial result [6].

Innovative treatments have emerged to further advance TMD management, notably through the exploration of non-pharmacologic methods, such as low-level laser therapy (LLLT) and transcutaneous electrical nerve stimulation (TENS), which have shown efficacy in pain reduction and improved joint function [7, 8]. Concurrently, tissue engineering represents an innovative development, offering the potential for the regeneration of temporomandibular joint tissues and providing sustainable long-term solutions [9]. Educational strategies, including advancements in diagnostic imaging, enhance the understanding and clinical outcomes of TMD [10], reflecting a trend towards more integrative and technology-driven methodologies.

DC/TMD is a standardized diagnostic framework that combines both physical (Axis I) and psychosocial (Axis II) aspects, despite considerable clinical variation. It was created to make the earlier RDC/TMD criteria more reliable and valid. The DC/TMD is highly accurate and reliable in diagnosing common pain-related TMDs, with good inter-examiner reliability. Its dual-axis structure supports the biopsychosocial model of TMD and allows categorization of disorders [11]. Consequently, TMD management protocols remain fragmented in their capacity to successfully translate diagnostic advancements into practical, outcome-focused treatment frameworks. Current clinical research uses scoping reviews to identify the possibilities for therapy [12]. The other study looks at the suggested limited algorithms, like the TMD algorithm, to help otolaryngologists figure out the best treatment options [13]. Previous studies have produced inconsistent outcomes due to dependence on generalized diagnostic categories, suggesting that a definitive, evidence-based treatment algorithm linked to specific diagnostic subtypes is still lacking.

Clinically, the first-line management of TMD often prioritizes conservative and reversible interventions, reserving invasive procedures for cases that are refractory. Systematic reviews have indicated advantageous effect sizes for pain relief, enhanced mouth opening, and functional improvement. However, the extent and duration of the effect can vary according to the type of intervention [14]. However, most of the literature and current reviews regard TMD as a uniform entity, with insufficient focus on subtype-specific responses. Over the past decade, substantial clinical research has evaluated innovative interventions for conservative and noninvasive treatments. However, the evidence remains fragmented, with varying outcome measures and follow-up durations. This scoping review of high-quality RCTs published between 2015 and 2025 is essential for systematically mapping the existing evidence, synthesizing comparative efficacy data, and distinguishing between treatments that primarily alleviate pain and those that achieve optimal clinical outcomes. The objectives of this study were to map the utilization of standardized axial diagnostic criteria and outcome measures in recent RCTs to synthesize the comparative efficacy and safety profiles of physical, pharmacological, appliance, and minimally invasive modalities for various TMD subtypes and to identify knowledge gaps and methodological limitations, thereby providing clinical implications for TMD management protocols.

## 2. Methods

### 2.1 Protocol

This scoping review was conducted in accordance with the PRISMA-ScR guidelines, as documented in the **Supplementary material 1** [15]. A review protocol was developed to define the objectives, eligibility criteria, and research methods. Below, we delineate the protocol elements, including the information sources, search strategy, study selection, data extraction, and synthesis approach, were registered in Open Science Framework (OSF) Registration: 10.17605/OSF.IO/VBD52.

### 2.2 Eligibility criteria

#### 2.2.1 Inclusion criteria

The inclusion criteria for this scoping review were developed using the Population–Concept–Context (PCC) framework, in accordance with the Joanna Briggs Institute guidance for scoping reviews and PRISMA-ScR recommendations. The PCC framework applied in this review is summarized in Table 1.

**Table 1.** PCC framework.

PCC Framework	Inclusion Criteria
Population	Studies involving human participants with a diagnosis of temporomandibular disorder including any TMD subtypes as defined by RDC/TMD, DC/TMD were eligible. Diagnostic subgroups comprised myofascial pain, myalgia, arthralgia, disc displacement with or without reduction, degenerative joint disease, headache attributed to TMD, and mixed TMD presentations. There were no age and no follow-up duration restrictions.
Concept	The Study focused on mapping and comparing evidence for diagnosis-based conservative and minimally invasive management pathways in TMD. Included interventions covered education, behavioral therapy, self-care programs, exercise-based physiotherapy, occlusal and stabilization splints, LLLT, TENS, ultrasound, PBMT, dry needling, acupuncture, arthrocentesis, and intra-articular injections. The main outcomes of interest were pain intensity, mandibular functional improvement, and psychological or quality-of-life parameters.
Context	Clinical and academic healthcare environments where TMD was managed according to recognized diagnostic frameworks. Only randomized controlled trials (RCTs) published between 2015 and 2025 were included to capture contemporary clinical practices. Studies were conducted across diverse geographic regions.

*TMD: Temporomandibular disorders; DC/TMD: Diagnostic Criteria for Temporomandibular Disorders; LLLT: low-level laser therapy; TENS: transcutaneous electrical nerve stimulation; RDC/TMD: Research Diagnostic Criteria for Temporomandibular Disorders; PBMT: Photobiomodulation Therapy.*

#### 2.2.2 Exclusion criteria

The exclusion Criteria for this review were case reports, editorials, systematic reviews, *in vitro* or animal studies, and trials focusing solely on complex surgical procedures or purely diagnostic imaging techniques. Studies involving patients with uncontrolled systemic diseases were excluded.

### 2.3 Information sources and search strategy

A comprehensive search was performed in four major databases: PubMed, Scopus, Web of Science, and Embase. The search strategy utilized a combination of controlled vocabulary Medical Subject Heading (MeSH) terms and free-text keywords to maximize sensitivity, available in Table 2 Search strategy.

**Table 2.** Search strategy with Boolean operator.

Database	Boolean Operator	Result
PubMed	((“Temporomandibular Joint Disorders”[MeSH Terms]) OR (“Temporomandibular Dysfunction Syndrome”) OR TMD) AND ((“Diagnosis”[MeSH Terms]) OR “DC/TMD” OR “RDC/TMD”) AND ((“Conservative Treatment”[MeSH Terms]) OR “Minimally Invasive” OR “Physical Therapy” OR “Occlusal Splint” OR “Photobiomodulation” OR “Laser Therapy” OR “Transcutaneous Electrical Nerve Stimulation” OR “Arthrocentesis” OR “Platelet-Rich Plasma”) AND (“Randomized Controlled Trial”[Publication Type] OR “RCT”) AND (2015:2025[pdat])	178
Scopus	(TITLE-ABS-KEY (“Temporomandibular Disorder*” OR “Temporomandibular Joint Disorder*” OR “TMD” OR “TMJ Disorder*” OR “Orofacial Pain”)) AND (TITLE-ABS-KEY (“Diagnosis” OR “DC/TMD” OR “RDC/TMD”)) AND (TITLE-ABS-KEY (“Conservative Treatment” OR “Non-surgical Management” OR “Physical Therapy” OR “Exercise” OR “Occlusal Splint” OR “Cognitive Behavioral Therapy” OR “Photobiomodulation” OR “Laser Therapy” OR “Transcutaneous Electrical Nerve Stimulation” OR “Arthrocentesis” OR “Platelet-Rich Plasma” OR “Hyaluronic Acid” OR “Needling”))	846
Embase	(’temporomandibular joint disorder’/exp OR ’temporomandibular dysfunction syndrome’ OR ’tmd’ OR ’tmj disorder’ OR ’orofacial pain’) AND (’diagnosis’/exp OR ’diagnostic criteria’ OR ’dc/tmd’ OR ’rdc/tmd’ OR ’research diagnostic criteria for temporomandibular disorders’) AND (’conservative treatment’/exp OR ’conservative therapy’ OR ’nonsurgical treatment’ OR ’noninvasive treatment’ OR ’minimally invasive’ OR ’physical therapy’/exp OR ’exercise therapy’/exp OR ’occlusal splint’ OR ’stabilization splint’ OR ’behavior therapy’/exp OR ’self- care’ OR ’patient education’/exp OR ’photobiomodulation’ OR ’low level laser therapy’/exp OR ’transcutaneous electrical nerve stimulation’/exp OR ’arthrocentesis’/exp OR ’platelet rich plasma’/exp OR ’hyaluronic acid’/exp OR ’needling’) AND (’randomized controlled trial’/exp OR ’clinical trial’/exp) NOT (’surgery’/exp OR ’arthroscopy’/exp) AND [english]/lim AND [humans]/lim AND [2015–2025]/py	251
Web of Science	TS = ((“temporomandibular disorder*” OR “temporomandibular joint disorder*” OR “temporomandibular dysfunction syndrome” OR “TMD” OR “TMJ disorder*” OR “orofacial pain”)) AND TS = (diagnos* OR “diagnostic criteria” OR “DC/TMD” OR “RDC/TMD” OR “research diagnostic criteria for temporomandibular disorders”) AND TS = (“conservative” OR “non-surgical” OR nonsurgical OR “minimally invasive” OR “physical therap*” OR exercise* OR “occlusal splint*” OR “stabilization splint*” OR “behavior* therap*” OR “behavio* therap*” OR “cognitive behavio* therap*” OR “self-care” OR “patient education” OR photobiomodulation OR “laser therap*” OR “low-level laser” OR TENS OR “transcutaneous electrical nerve stimulation” OR arthrocentesis OR “platelet-rich plasma” OR “hyaluronic acid” OR needling OR “dry needling”) AND TS = ((random* NEAR/2 (trial* OR control* OR assign* OR allocat*)) OR “randomized controlled trial” OR “randomised controlled trial” OR “clinical trial”) NOT TS = (surg* OR arthroscop*)	214

*DC/TMD: Diagnostic Criteria for Temporomandibular Disorders; RDC/TMD: Research Diagnostic Criteria for Temporomandibular Disorders; TMJ: Temporomandibular Joint; MeSH: Medical Subject Heading; TITLE-ABS-KEY: Title-Abstract-Keyword.*

### 2.4 Study selection

Records were exported to Rayyan, where duplicates were eliminated using automated deduplication and manual verification processes. The study selection process is illustrated in a PRISMA flow diagram. Two independent reviewers (GZ, AM) screened the titles and abstracts, evaluating each record for relevance to TMD and adherence to the inclusion criteria. A liberal inclusion approach was employed, in which the full text was obtained if either reviewer deemed a citation to be potentially eligible. Studies not pertinent to TMD, including those that focused exclusively on temporomandibular anatomy or alternative conditions, were excluded. The complete texts of the remaining articles were obtained and assessed independently by two reviewers based on the eligibility criteria, with reasons for exclusion documented. Discrepancies among the reviewers were addressed through discussion and consensus or by the inclusion of a third reviewer (HM). When full texts were not accessible despite meeting our “full text available” criteria, we pursued inter-library loans and reached out to the authors. Several full texts were inaccessible and were thus excluded from consideration. A consensus approach was used for the final inclusion decisions.

### 2.5 Data extraction

We created a standardized data extraction form in a spreadsheet to systematically record the data from the included studies. Two reviewers conducted a pilot test utilizing five studies and implemented changes. The data that was taken out comprised bibliographic information about the research (author(s), year of publication, place of origin), information about the population (sample size, age, sex distribution, patient details), and the diagnostic framework utilized (RDC/TMD or DC/TMD). We tracked the interventions examined in each study, including all arms for controlled trials and information on the dose, frequency, and length of treatment. Thereafter, the interventions were grouped. We gathered clinical outcomes, including pain, function, and psychological metrics. We summarized the most important results of each trial, highlighting how much better the interventions were than the controls and writing down the authors’ conclusions. One reviewer performed the first data extraction, while a second reviewer confirmed the data. We were able to clarify any differences by examining the entire text.

### 2.6 Risk of bias assessment

The methodological quality of the included randomized controlled trials were evaluated using the Cochrane Risk of Bias 2.0 tool, the current gold standard instrument for assessing bias in randomized studies. Two reviewers independently assessed each study across five domains: bias arising from the randomization process, deviations from the intended interventions, missing outcome data, outcome measurement, and selection of reported results. Disagreements were resolved through discussion or consultation with a third reviewer, if necessary. Judgments were categorized as “low risk”, “some concerns”, or “high risk” according to the Version 2 of the Cochrane risk-of-bias tool for randomized trials (RoB2) algorithm.

## 3. Result

### 3.1 Study selection

A literature search across the four databases identified 1454 records after removing duplicates (Fig. 1). After the initial duplication of 297 records, 1157 unique citations remained for screening purposes. We screened the titles and abstracts of these 1157 articles, excluding 1007 irrelevant or ineligible records; most were unrelated to TMD or were not clinical trials. This resulted in 150 articles for full-text retrieval and evaluation. Of these 150, we were unable to obtain 15 full texts despite extensive efforts, because none of the authors replied to these requests, any studies without accessible full texts or key data were excluded, full PDFs were not accessible and thus were excluded. We assessed the remaining 135 full-text articles in detail; 6 studies were excluded at this stage because they did not meet the eligibility criteria. This scoping review included 129 studies that met all the inclusion criteria.

**Fig. 1.** PRISMA flow diagram. The flow diagram depicts the number of records identified, screened, and included in this study. DC: Diagnostic Criteria; RDC: Research Diagnostic Criteria.

### 3.2 Characteristics of included studies

The 129 included RCTs demonstrated a wide geographical distribution, with prominent representations from Asia, Europe, and America. All included studies were randomized controlled trials. These trials were published between 2015 and 2025, reflecting the last decade of research. The sample sizes ranged from relatively small trials of approximately 16 participants to larger trials with nearly 202 participants, with a median sample size of 48 patients. Most studies enrolled young to middle-aged adults with a mean age in the 30s, and overwhelmingly female participants (>80% women on average). See **Supplementary Table 1** Study Characteristics.

Most studies were single-center trials, although a few were multicenter or multicountry collaborations. Geographic mapping revealed that research on temporomandibular disorder (TMD) management originated from Brazil, Canada, Europe (including Sweden, Germany, and Croatia), Asia (Including Turkey, China, and India), and the Middle East (including Iraq and Saudi Arabia). Notably, Brazil, with 33 studies of origin, was the most common, followed by Turkey, India, and various European nations.

Most studies utilized established standardized frameworks, either the RDC/TMD or the DC/TMD. A total of 57 RCTs used DC/TMD, while 72 used older RDC/TMD. The adoption of DC/TMD was associated with more precise subtype stratification, especially in distinguishing myofascial pain from arthralgia. Studies using multi-axial frameworks were more likely to incorporate Axis II psychosocial assessments and reported better alignment between diagnosis and targeted intervention outcomes. See **Supplementary Table 2** Data Extraction.

### 3.3 Thematic synthesis: diagnosis-based treatment pathways

A total of 129 RCTs were identified that examined the various interventions. These interventions were classified into first-line conservative therapies and minimally invasive treatments, following the treatment hierarchy adopted across the included studies. Conservative management approaches, such as Physical Exercise, Behavioral Therapy, Self-Care, Physical Therapy and Exercise, Oral Splint Therapy, Physical Modalities, and Pharmacotherapy, are considered first-line therapies. When conservative approaches failed or resulted in limited improvement, minimally invasive interventions, including Arthrocentesis/Joint Lavage, Injection, and Needling, were considered. See Table 3 (Ref. [16, 17, 18, 19, 20, 21, 22, 23, 24, 25, 26, 27, 28, 29, 30, 31, 32, 33, 34, 35, 36, 37, 38, 39, 40, 41, 42, 43, 44, 45, 46, 47, 48, 49, 50, 51, 52, 53, 54, 55, 56, 57, 58, 59, 60, 61, 62, 63, 64, 65, 66, 67, 68, 69, 70, 71, 72, 73, 74, 75, 76, 77, 78, 79, 80, 81, 82, 83, 84, 85, 86, 87, 88, 89, 90, 91, 92, 93, 94, 95, 96, 97, 98, 99, 100, 101, 102, 103, 104, 105, 106, 107, 108, 109, 110, 111, 112, 113, 114, 115, 116, 117, 118, 119, 120, 121, 122, 123, 124, 125, 126, 127, 128, 129, 130, 131, 132, 133, 134]) Diagnosis-Based Treatment Pathways.

**Table 3.** Diagnosis-based treatment pathways.

Axis I Diagnosis	No. of RCTs	First-Line Conservative Therapies		Minimally Invasive Intervention	
Myofascial Pain	48	1. PE/BT/SC [16, 17, 18, 19, 20, 21, 22, 23, 24]		1. Needling	● Dry Needling [25, 30, 31, 32, 33, 34] ● Auricular Acupuncture [35]
		2. Physical Therapy & Exercises	● Manual Therapy [22, 23, 25, 26, 27] ● Jaw exercises [19, 28] ● Kinesio Taping (KT) [24, 29]	2. Injections	● Saline [42] ● Collagen MD Muscle [42] ● Lidocaine [43]
		3. Oral Splints	● RehaBite® [36] ● Michigan splint [16, 36, 37, 38] Interocclusal Splint Therapy [25] Stabilization splint [18, 19, 20, 21, 39, 40, 41]		
		4. Physical Modalities	● LLLT [23, 38, 44, 45, 46, 47, 48, 49, 50] ● TENS [41, 44, 51, 52, 53, 54, 55] ● PBMT ● Red Light Therapy [45, 56] ● ELIBA [57] ● aVNS/taVNS [58]		
		5. Pharmacotherapy	● Diclofenac Sodium [25, 40] ● Vitamin D [40] ● Paracetamol [33] ● Other NSAIDs [17]		
Myalgia	26	1. PE/BT/SC [21, 59, 60, 61, 62]		1. Needling	● Acupuncture [67]
		2. Physical Therapy & Exercises [61, 63, 64]	● Manual Therapy	2. Injections	● Saline [74, 75] ● BoNT/A [74, 75] ● EGF [76]
		3. Oral Splints (OAT)	● Stabilization Splint [39, 65] ● CAD/CAM Splint [66] ● Thermoformed Splint [60]		
		4. Physical Modalities	● LLLT [68, 69] ● PSWT [70] ● Ultrasound [71, 72] ● HILT [72] ● rTMS [73]		
		5. Pharmacotherapy	NSAID & Relaxant [70]		
Arthralgia	30	1. PE/BT/SC [21, 59, 61, 62, 77, 78]		1. Arthrocentesis/Joint Lavage	● Saline [81] ● Ringer Lactate [82] ● Methylprednisolone [82] ● HA [83, 84] ● PRP [84] ● Local Anesthetic [77, 81]
		2. Physical Therapy & Exercises	● Manual Therapy [59, 77, 79, 80] ● Jog Manipulation [61] ● Jaw Exercise [28]	2. Injections	● Saline [74, 75, 87] ● BoNT/A [74, 75] ● Methylprednisolone [82, 87] ● Local Anesthetic [81] ● Betamethasone [86] ● HA [82, 84, 86, 88] ● PRP [83, 88] ● Triamcinolone [88] ● Viscosupplementation [89]
		3. Oral Splints	● CAD/CAM Splint [66] ● Occlusal Splint [21, 85, 86] ● Michigan [21] & Stabilization Splint [28, 39, 80]	3. Needling	● Acupuncture [91] ● Dry Needling [92]
		4. Pharmacotherapy	● Glucosamine hydrochloride, chondroitin sulfate, methylsulfonylmethane [84] ● Tenoxicam [77] ● Amitriptyline [90]		
Disc Displacement with Reduction (DDR)	17	1. PE/BT/SC [93, 94, 95, 96]		1. Injections	● Saline [99] ● Dextrose [99] ● BoNT/A [97, 100]
		2. Physical Therapy & Exercises [94, 95, 96]	● Manual Therapy		
		3. Oral Splints	● ARS [93, 97] ● Occlusal Splint [96, 98]		
		4. Physical Modalities	● LLLT [97, 98] ● US [93]		
		5. Pharmacotherapy	● Propranolol [101]		
Disc Displacement without Reduction (DDWoR)	15	1. PE/BT/SC [61, 77, 102, 103]		1. Arthrocentesis/Joint Lavage	● Conventional [77, 102, 104] ● Single Puncture [105, 106] ● Double Puncture [105, 106, 107] ● US Guided [108] ● HA [109] ● PRP [109]
		2. Physical Therapy & Exercises	● Physiotherapy [77] ● Jog Manipulation [61]		
		3. Oral Splints	● ARS [103] ● CAD/CAM Splint [66] ● Occlusal splint [102, 104]		
		4. Physical Modalities	● LLLT [110] ● TENS [110]		
Degenerative Joint Disease (DJD)	7	1. PE/BT/SC [59, 77, 78, 103]		1. Arthrocentesis/Joint Lavage	● Conventional [77] ● HA [78]
		2. Physical Therapy & Exercises [59, 77]	● Manual Therapy	2. Injection	● Saline [75] ● BoNT/A [75]
		3. Oral Splints	● ARS [103]		
		4. Physical Modalities	● Pulsed Radiofrequency [78] ● LLLT [110] ● TENS [110]		
		5. Pharmacotherapy	● Glucosamine [111]		
Headache Attributed to TMD	4	1. PE/BT/SC [78]		1. Injections	● Saline [74, 75, 112] ● BoNT/A [74, 75, 112]
		2. Physical Modalities	● Pulsed Radiofrequency [78]		
Mixed TMD	22	1. PE/BT/SC [113, 114, 115, 116, 117, 118]		1. Arthrocentesis/Joint Lavage [122]	● Conventional Arthocentesis
		2. Physical Therapy and Exercises	● Physiotherapy [114, 119] ● Manual Therapy [27, 114, 115, 118, 120] ● Bowen’s [121]	2. Needling	● Scalp Acupuncture [115] ● Ear Acupuncture [128]
		3. Oral Splints	● Occlusal Splint [113, 115, 116, 117, 118, 123, 124] ● BFB Splint [125] ● Michigan splint [126] ● SOVA Splint [126] ● Stabilization Splint [127]	3. Occlusal Equilibration Therapy [119, 134]	
		4. Physical Modalities	● LLLT [119, 129, 130, 131] ● TENS [27, 119, 120, 131, 132] ● Ozone [123] ● PBMT [133] ● US [121]		
		5. Pharmacotherapy	● Duloxetine [122]		

*BoNT/A: Botulinum toxin A; DJD: Degenerative joint disease; DDR: Disc displacement with reduction; DDWoR: Disc displacement without reduction; HA: Hyaluronate; LLLT: Low-level laser therapy; OAT: Oral appliance therapy; PE: Patient education; BT: Behavioral therapy; SC: Self-care; PE/BT/SC: Patient education/behavioral therapy/self-care; PRP: Platelet-rich plasma; PSWT: Pulsed short-wave therapy; TENS: Transcutaneous electrical nerve stimulation; TMD: Temporomandibular disorders; KT: Kinesio Taping; PBMT: Photobiomodulation therapy; ELIBA: Lingual Elevation by Induced Biofeedback Appliance; aVNS/taVNS: Auricular Vagus Nerve Stimulation/Transcutaneous Auricular Vagus Nerve Stimulation; NSAIDs: Non-steroidal anti-inflammatory drugs; CAD/CAM: Computer-aided design/Computer-aided manufacturing; HILT: High-intensity laser therapy; EGF: Epidermal Growth Factor; US: Ultrasound; ARS: Anterior Repositioning Splint; BFB: Biofeedback; SOVA: SOVA dental splint; BFB Splint: Biofeedback Splint; RCTs: Randomized Controlled Trials; rTMS: Repetitive transcranial magnetic stimulation.*

#### 3.3.1 Management of myofascial pain

First-line management emphasizes patient education/behavioral therapy/self-care (PE/BT/SC), which significantly improved pain and function [16, 17, 18, 19, 20, 21, 22, 23, 24]. The integration of physical therapy and jaw exercises further enhances mandibular mobility [23, 26, 28]. Kinesio taping and Oral Splint training improves coordination and muscle endurance [24, 36]. Oral splints, particularly Michigan and stabilization types, produced consistent analgesic and psychosocial benefits [37, 39]. Among the physical modalities, LLLT, TENS, and Photobiomodulation Therapy (PBMT) achieved measurable pain and functional gains [45, 47, 51, 54]. Red light therapy, neuromuscular lingual device ELIBA® (lingual elevator, Balercia Company, Ancona, Italy), and auricular vagus nerve stimulation have shown promising neuromodulatory effects [56, 58]. Pharmacotherapy with diclofenac, vitamin D, and paracetamol offers supportive, short-term relief [40]. For refractory cases, dry needling and auricular acupuncture (AA) effectively reduce trigger-point pain and improve sleep quality [25, 31, 35]. Injections of saline, collagen MD muscle, or lidocaine also provided transient pain relief [42, 43].

#### 3.3.2 Management of myalgia

Primary approaches combined PE/BT/SC and exercise-based physiotherapy, which improved muscle endurance and mobility [59, 61, 62, 63, 64]. Stabilization, computer-aided design/computer-aided manufacturing (CAD/CAM), and thermoforming splints provide comparable benefits [39, 65, 66]. Physical modalities, including LLLT, high-intensity laser therapy (HILT), pulsed shockwave therapy (PSWT), and ultrasound (US), produced significant analgesia and functional recovery [68, 70, 71, 72]. nonsteroidal anti-inflammatory drugs (NSAIDs) and muscle relaxants act as adjuncts to physiotherapy [70]. For persistent myalgia, acupuncture and botulinum toxin type A (BoNT/A) achieved additional pain relief, whereas EGF injections showed early promise [67, 74, 75, 76].

#### 3.3.3 Management of arthralgia

Conservative interventions that combine education, behavioral therapy, and physiotherapy improve joint function and psychosocial outcomes [59, 77, 78]. Manual therapy, jog-manipulation, and jaw exercises reduced pain and enhanced mandibular range [61, 79]. Stabilization and CAD/CAM splints yielded biochemical and symptomatic improvements [39, 80]. When conservative measures plateaued, arthrocentesis with saline or Ringer’s lactate, optionally followed by hyaluronic acid (HA) or platelet rich fibrin (PRP), achieved greater pain relief and mouth-opening gains [82, 85, 88]. Corticosteroids, betamethasone, and local anesthetics provided transient benefit [86, 87]. Acupuncture and dry needling also enhanced oxygenation and pain modulation [91, 92]. Pharmacologic agents such as amitriptyline and glucosamine/chondroitin further supported chronic pain control [84, 90].

#### 3.3.4 Management of disc displacement with reduction (DDR)

PE/BT/SC with jaw exercises improved clicking and functional limitation [94, 95]. Anterior repositioning splint (ARS) demonstrated superior improvements in pain and disc position [93, 96]. LLLT, US, and arthrocentesis expedited pain relief [98]. BoNT/A and dextrose prolotherapy were effective adjuncts [99, 100]. Propranolol reduced sympathetic-linked pain intensity without major adverse effects [101].

#### 3.3.5 Management of disc displacement without reduction (DDWR)

Conservative therapy including education, physiotherapy, and jog-manipulation improved mandibular mobility and reduced pain [61, 77]. Arthrocentesis performed conventionally or with single/double-puncture or US guidance, achieved comparable success [102, 106, 108]. HA and PRP injections provided longer-lasting relief than lavage alone [109]. ARS and occlusal or CAD/CAM splints supported joint realignment [66, 103]. LLLT and TENS delivered additional analgesia [110].

#### 3.3.6 Management of degenerative joint disease (DJD)

Education, cognitive behavior therapy (CBT), and exercise therapy improved mandibular kinematics [59, 77]. ARS splints enhanced condylar regeneration in early-stage cases [103]. Arthrocentesis with HA effectively reduced pain [111]. Pulsed radiofrequency, LLLT, and TENS provided neuromodulatory benefits [78, 110], while oral glucosamine promoted condylar repair [111].

#### 3.3.7 Management of headache attributed to TMD

PE/BT/SC and pulsed radiofrequency yielded consistent pain reduction [78]. BoNT/A injections improved headache frequency and intensity compared with saline, though results were variable [74, 75, 112].

#### 3.3.8 Management of mixed temporomandibular disorders

Integrated multimodal conservative management combining education, self-care, splints, and manual or cervical physiotherapy produced broad improvements in pain, function, and sleep quality [113, 114, 115, 117]. Biofeedback Splint (BFB) and SOVA splints reduced bruxism and muscle hyperactivity [125, 126]. Physical modalities such as LLLT, TENS, ozone, and PBMT achieved additional analgesic benefit [123, 131, 133]. Acupuncture, occlusal equilibration, and arthrocentesis plus duloxetine were effective adjuncts for persistent pain [122, 128, 134].

### 3.4 Overview of outcome measures

The analysis of outcome measures across the 129 included studies reveals a strong focus on self-reported pain intensity and basic functional mobility, including complex quality of life (QoL) and objective physiological markers. The most frequently reported outcome was the VAS, which was utilized in 84 studies (65%). This reliance on simple, unidimensional pain assessment was followed by the Numerical Rating Scale (NRS) in 22 studies (17%), while more holistic pain instruments like the Graded Chronic Pain Scale (GCPS) and Characteristic pain intensity (CPI) were each found in only 8 studies (6%). In terms of functional mobility, MMO was highly prevalent, used in 58 studies (45%), second only to VAS in overall frequency, while the Jaw Functional Limitation Scale-20 (JFLS) and “Jaw function” were each reported in 12 and 11 studies respectively (9% each). Psychological and QoL measures were also included, underscoring the recognized biopsychosocial nature of TMD. For QoL assessment, general QoL measures were found in 17 studies (13%) and the Oral Health Impact Profile-14 (OHIP-14) in 9 studies (7%). Psychological status was assessed with the Patient Health Questionnaire-9 (PHQ-9) was used in 5 studies (4%), while the Generalized Anxiety Disorder-7 (GAD-7) and Beck Depression Inventory (BDI) were each found in 4 studies (3%). Finally, objective and physiological measures show an emerging trend, with Pressure pain threshold (PPT) measured in 15 studies (12%) and Surface electromyography (sEMG) in 14 studies (11%), suggesting an interest in quantifying local tissue sensitivity and muscle activity. Specific biomarker analysis remains scarce, only appearing as single instances of Salivary Cortisol (1%) and Oxidative Stress Markers (1%), along with Interleukin-6 (IL-6) (1%), each in 1% of the studies. See Table 4 Outcome Measure.

**Table 4.** Outcome measures.

Outcome Domain	Outcome Measure	Frequency in RCTs
Pain Intensity		
	VAS	84 (65%)
	NRS	22 (17%)
	CPI	8 (6%)
	GCPS	8 (6%)
	BPI	1 (1%)
Functional Mobility		
	MMO	58 (45%)
	JFLS	12 (9%)
	Jaw function	11 (9%)
	Bite force	2 (2%)
	UMO	1 (1%)
	CLM	1 (1%)
Psychological/Emotional		
	PHQ-9	5 (4%)
	GAD-7	4 (3%)
	BDI	4 (3%)
	PCS	4 (3%)
	HADS	3 (2%)
	DASS-21	1 (1%)
	PSEQ	1 (1%)
Quality of Life/Well-being		
	QoL	17 (13%)
	OHIP-14	9 (7%)
	PGIC	7 (5%)
	PSQI	4 (3%)
	GPE	1 (1%)
	SF-36	1 (1%)
Physiological/Objective		
	PPT	15 (12%)
	sEMG	14 (11%)
	IL-6	1 (1%)
	Oxidative stress	1 (1%)
	Salivary cortisol	1 (1%)
	Trismus	1 (1%)

*VAS: Visual Analogue Scale; NRS: Numeric Rating Scale; PPT: Pressure Pain Threshold; MMO: Maximum Mouth Opening; UMO: Unassisted Mouth Opening; CLM: Condylar-Lateral Mandibular Movement; JFLS: Jaw Functional Limitation Scale; GCPS: Graded Chronic Pain Scale; BPI: Brief Pain Inventory; CPI: Chronic Pain Intensity; BDI: Beck Depression Inventory; DASS-21: Depression, Anxiety, and Stress Scale-21 items; GAD-7: Generalized Anxiety Disorder-7; HADS: Hospital Anxiety and Depression Scale; PHQ-9: Patient Health Questionnaire-9; PCS: Pain Catastrophizing Scale; PSEQ: Pain Self-Efficacy Questionnaire; OHIP-14: Oral Health Impact Profile-14; PSQI: Pittsburgh Sleep Quality Index; QoL: Quality of Life; SF-36: Short Form Health Survey-36; GPE: Global Perceived Effect; PGIC: Patient Global Impression of Change; EMG: Electromyography; IL-6: Interleukin-6; RCTs: randomized controlled trials.*

### 3.5 Risk of bias assessment

Of the 129 RCTs evaluated, most had low to moderate methodological quality (Fig. 2). According to RoB2, 34% were rated as low risk, 52% had some concerns, and 14% were high risk. Common issues involved unclear allocation concealment (41%), lack of blinding (58%, especially for physical treatments), and deviations from interventions due to inconsistent adherence (37%). Outcome measurement bias was low, with validated scales used widely, while selective reporting was uncommon (89% reported all prespecified outcomes). Comprehensive RoB2 assessments are presented in Table 5 Risk of Bias Judgement by RoB2.

**Fig. 2.** RoB2 summary.

**Table 5.** Risk of bias judgement by RoB2.

Study	D1	D2	D3	D4	D5	Overall
Abbasgholizadeh, 2020	Low	Some concerns	Low	Low	Low	Some concerns
Aguiar, 2023	Low	High	Low	Low	Low	High
Aisaiti, 2021	Low	Low	Some concerns	Low	Low	Some concerns
Al-Quisi, 2023	Low	Low	Low	Low	Low	Low
Alajbeg IZ, 2020	Low	Low	Some concerns	Low	Low	Low
Anupriya, 2023	Low	Some concerns	Some concerns	Low	Low	Some concerns
Aroca, 2022	Low	Low	Low	Low	Low	Low
Babiloni, 2024	Low	Low	Low	Low	Some concerns	Some concerns
Baker, 2015	Low	Low	Some concerns	Low	Low	Low
Barbosa, 2019	Low	Some concerns	Low	Low	Low	Low
Benli, 2021	Low	Some concerns	Low	Low	Low	Low
Bergmann, 2020	Low	High	Some concerns	Low	Low	High
Bhargava, 2019	Some concerns	Some concerns	Low	Some concerns	Low	Some concerns
Bijelic, 2024	Low	High	High	Low	Low	High
Brakus, 2025	Low	Low	High	Low	Some concerns	High
Brochado, 2018	Some concerns	Some concerns	Low	Low	Low	Some concerns
Cattaneo, 2019	Some concerns	Some concerns	Some concerns	Low	Some concerns	Some concerns
Celakil, 2017	Low	Some concerns	Low	Low	Low	Low
Chami, 2020	Low	Low	Some concerns	Low	Some concerns	Some concerns
Costa, 2015a	Low	High	Some concerns	Low	Low	Some concerns
Costa, 2015b	Low	Some concerns	Some concerns	Low	Low	Some concerns
Costa, 2017	Low	Low	Low	Low	Low	Low
Darwin, 2024	Some concerns	High	Low	Some concerns	Some concerns	High
De Nordenflycht, 2022	Low	Some concerns	Low	Low	Low	Some concerns
De Nordenflycht, 2024	Low	Some concerns	Low	Low	Low	Some concerns
Delgado de la Serna, 2019	Low	Some concerns	Low	Some concerns	Low	Some concerns
Deregibus, 2021	Low	High	Low	High	Some concerns	High
Dhanasekaran, 2022	Some concerns	High	Some concerns	Some concerns	Some concerns	Some concerns
Doepel, 2017	Some concerns	Some concerns	Some concerns	Some concerns	Some concerns	Some concerns
Dunning, 2024	Low	Some concerns	Low	High	Low	High
Ekici, 2022	Some concerns	Some concerns	Low	Low	Low	Some concerns
Ferreira, 2017	Low	Some concerns	High	Low	Low	Some concerns
Folle, 2018	Low	Some concerns	Low	Low	Some concerns	Some concerns
Furquim, 2023	Low	High	Low	Low	Low	High
García, 2023	Low	High	Low	Some concerns	Low	High
Gębska, 2023	Low	Some concerns	Low	Some concerns	Low	Some concerns
Gębska, 2024	Low	Some concerns	Low	Low	Low	Some concerns
Gerstner, 2020	Low	High	Low	Low	Low	Some concerns
Giannakopoulos, 2016	Low	High	Low	Low	Low	High
Giannakopoulos, 2018	Low	High	Some concerns	Low	Low	Some concerns
Gikić, 2021	Low	High	Some concerns	Low	Some concerns	Some concerns
Giro, 2016	Some concerns	Some concerns	High	Low	Low	High
Godoy, 2017	Low	Some concerns	Low	Low	Low	Low
Gonzalez-Perez, 2015	Low	High	Some concerns	Low	Low	Some concerns
Grossmann, 2017	Low	Low	Low	Low	Low	Low
Grossmann, 2021	Low	Some concerns	Low	Low	Low	Some concerns
Guarda-Nardini, 2015	Low	Some concerns	Low	Low	Low	Some concerns
Haggag, 2022	Low	Low	Low	Low	Low	Low
Hasanoglu Erbasar, 2017	Low	Some concerns	Low	Low	Low	Low
Herpich, 2018	Low	Low	Low	Low	Low	Low
Herpich, 2020	Low	Low	Low	Low	Low	Low
Huttunen, 2018	Some concerns	High	High	Some concerns	Low	High
Hwangbo, 2023	Some concerns	Some concerns	Some concerns	Some concerns	Some concerns	Some concerns
Isacsson, 2019	Low	Some concerns	Some concerns	Low	Low	Some concerns
Javed, 2024	Low	High	Low	Low	Low	Some concerns
Jo, 2021	Low	Low	Some concerns	Low	Low	Some concerns
Kahraman, 2025	Low	Some concerns	Some concerns	Some concerns	Some concerns	Some concerns
Kiliç, 2016a	Some concerns	Some concerns	Low	Some concerns	Low	Some concerns
Kiliç, 2016b	Low	Some concerns	Low	Low	Low	Low
Kılıç, 2021	Low	Some concerns	Low	Some concerns	Low	Some concerns
Kopacz, 2020	Low	Some concerns	Low	Low	Low	Low
Kutuk, 2019	Low	High	Some concerns	Some concerns	Low	High
Lei, 2019	Low	Some concerns	Low	Low	Low	Some concerns
Leite, 2020	Low	Some concerns	Low	Low	Low	Some concerns
Lennartsson, 2025	Low	Low	Low	Low	Low	Low
Li, 2024	Low	High	High	High	Low	High
Lindfors, 2020	Low	High	Some concerns	Low	Low	High
Liu L, 2024	Low	Low	Low	Low	Low	Low
Macedo de Sousa, 2025	Low	High	Low	Low	Low	Some concerns
Macedo, 2020	Low	Some concerns	Low	Low	Low	Low
Macedo, 2023	Low	Low	Low	Low	Low	Low
Machado, 2016	Low	Some concerns	Some concerns	Low	Low	Some concerns
Madani, 2019	Low	Low	Low	Low	Some concerns	Low
Magri, 2017	Low	Low	Some concerns	Low	Low	Low
Magri, 2019	Low	Low	High	Low	Low	High
Majid, 2020	Low	High	Some concerns	Low	Low	Some concerns
Malekzadeh, 2019a	Low	High	Low	Some concerns	Low	High
Malekzadeh, 2019b	Low	High	Low	Low	Low	Some concerns
Maracci, 2020	Low	High	Low	Low	Low	Some concerns
Monteiro, 2020	Low	Low	Low	Low	Low	Low
Moraes, 2022	Low	Low	Some concerns	Low	Low	Low
Nadershah, 2019	Low	Low	Low	Low	Low	Low
Nagata, 2015	Low	High	Low	Low	Low	High
Nagata, 2019	Low	Some concerns	Low	Low	Low	Low
Nemani, 2024	Low	Low	Low	Low	Low	Low
Nitecka-Buchta, 2018	Low	Some concerns	Low	Low	Some concerns	Some concerns
Oliveira-Souza, 2024	Low	Low	Low	Low	Low	Low
Corrêa-Silva, 2024	Some concerns	Some concerns	Some concerns	Some concerns	Some concerns	Some concerns
Özden, 2018	Some concerns	High	Some concerns	Some concerns	Low	High
Packer, 2015	Low	Some concerns	Low	Low	Low	Some concerns
Patil, 2017	Low	Some concerns	Low	Some concerns	Low	Some concerns
Patil, 2023	Some concerns	Some concerns	High	Some concerns	Some concerns	High
Peixoto, 2021	Low	High	Low	Low	Low	Some concerns
Percin, 2025	Low	Some concerns	Some concerns	Low	Low	Some concerns
Pho Duc, 2016	Low	Low	High	Some concerns	Low	High
Pihut, 2018	Low	Some concerns	Low	Low	Low	Low
Pihut, 2020	Some concerns	Low	Some concerns	Low	Low	Some concerns
Polat, 2021	Low	Some concerns	Some concerns	Low	Low	Some concerns
Prati, 2024	Low	Some concerns	Low	Low	Low	Low
Qataya, 2025	Low	High	Some concerns	Low	Low	High
Rady, 2022	Low	High	Low	Low	Low	Some concerns
Ram, 2021	Low	Some concerns	Some concerns	Low	Some concerns	Some concerns
Resende, 2019	Some concerns	Some concerns	Some concerns	Low	Low	Some concerns
Rezaie, 2022	Low	Some concerns	Some concerns	Low	Low	Some concerns
Rezazadeh, 2022	Low	Low	Low	Low	Low	Low
Ritto, 2022	Low	Low	Some concerns	Low	Low	Low
Rodriguez-Blanco, 2015	Low	Low	Low	Low	Low	Low
Rodríguez CI, 2024	Low	High	Low	Some concerns	Low	High
Saglam, 2024	Some concerns	High	Some concerns	Low	Low	High
Saini, 2025	Low	Low	Low	Low	Low	Low
Sajedi, 2022	Low	Low	Low	Low	Low	Low
Sancakli, 2015	Low	Some concerns	Some concerns	Low	Low	Some concerns
Santana-Penín, 2023	Low	Low	Low	Low	Low	Low
Şen, 2020	Low	Some concerns	Some concerns	Low	Low	Some concerns
Simões, 2023	Low	Some concerns	Low	Low	Low	Some concerns
Singh, 2017	Low	High	Low	Low	Low	High
Siqueira, 2025	Low	High	Some concerns	Low	Low	High
Sitnikova, 2024	Low	Low	Low	Low	Low	Low
So Ra Kim, 2023	Low	Low	Low	Low	Low	Low
Taleb, 2025	Low	Low	Low	Low	Low	Low
Tchivileva, 2020	Low	Low	Low	Low	Low	Low
Toameh, 2019	Low	High	Low	Low	Low	Some concerns
van Grootel, 2017	Low	Low	Low	Low	Low	Low
Wahlund, 2017	Some concerns	High	Some concerns	High	Low	High
Wahlund, 2021	Low	Low	Some concerns	Low	Low	Low
Wänman, 2019	Low	Some concerns	Low	Low	Low	Low
Yang, 2018	Low	Low	Low	Low	Low	Low
Yeladandi, 2024	Some concerns	Some concerns	Low	Some concerns	Low	Some concerns
Zotelli, 2017	Low	Some concerns	Low	Low	Low	Low

## 4. Discussion

### 4.1 Summary of key findings

This scoping review systematically mapped and synthesized 129 RCTs to identify diagnosis-based treatment pathways for TMD using the DC/TMD & RDC/RMD framework, focusing on the progression from conservative to minimally invasive interventions and their corresponding outcomes. Overall, the findings demonstrate that education, behavioral therapy, and exercise-based rehabilitation remain the cornerstone of first-line management across all TMD diagnostic subtypes, producing consistent improvements in pain and mandibular function [16, 22]. The integration of splint therapy and physical modalities such as LLLT, TENS, US, and PBMT enhanced pain modulation and muscular relaxation [51, 54, 79]. Recent studies have affirmed that conservative therapy remains the primary choice in the management of TMD [135, 136, 137]. Minimally invasive procedures including dry needling, acupuncture, arthrocentesis, and intra-articular injections with HA, PRP, or BoNT/A were typically reserved for refractory or chronic cases and demonstrated additional, though variable, benefits in long-term pain control and joint function [75, 82, 103]. These results reinforce a stepwise, multimodal approach consistent with patient-centered management principles, in which conservative modalities are prioritized before escalating to invasive therapies, Notably, a single treatment does not fit all TMD cases; multidisciplinary approaches are proven most effective [138]. Across all diagnostic groups, VAS-based pain reduction and MMO improvement emerged as the dominant outcome measures, reflecting both symptomatic and functional recovery [96, 139].

#### 4.1.1 Management of myofascial pain

Myofascial pain remains the most investigated TMD subtype. PE/BT/SC with physiotherapy particularly soft-tissue manual therapy, cervical techniques, and jaw exercises enhances mandibular mobility, decreases masseter hyperactivity, and improves muscular endurance, findings corroborated by broader systematic reviews demonstrating moderate, clinically meaningful effects for craniomandibular manual therapy [14]. Kinesio taping and structured exercise programs enhance proprioception and coordination, supporting improvements in functional patterns [140, 141]. Oral appliances such as Michigan splints, stabilization splints, interocclusal devices, and RehaBite® yield variable but meaningful reductions in pain-related disability and oxidative stress, aligning with meta-analytic evidence showing that stabilization splints offer short-term analgesic benefits comparable to other conservative modalities [142]. Physical modalities including PBMT, red-light therapy, LLLT, PBMT, TENS, ELIBA neuromuscular training, and Auricular/Transcutaneous Auricular Vagus Nerve Stimulation (aVNS/taVNS) effectively alleviated pain and enhanced MMO without causing adverse effects, consistent with meta-analytic indicating that laser therapy offers small-to-moderate pain relief [143]. This modalities help in neuromodulation and decrease EMG activity, with reduced muscle activity at rest and sometimes better activation during voluntary movement. with measurable yet modest effects, consistent with updated laser-therapy reviews showing small-to-moderate pain relief [143]. Pharmacologic agents such as diclofenac, NSAIDs, paracetamol, and vitamin D supplementation provide short-term symptomatic relief and may benefit patients with low vitamin D levels [6, 40].

Dry needling consistently reduces trigger-point hyperalgesia, increases pain-free mouth opening [144], and other study improves sleep and tinnitus-associated symptoms mirroring meta-analytic data demonstrating its neuromodulatory effects in chronic musculoskeletal pain [145]. Auricular acupuncture also reduces pain and improves global function, with effects comparable to occlusal splints in RCTs, and supported by systematic reviews documenting acupuncture’s efficacy in myogenous TMD [146, 147]. Injections of saline, collagen MD, or lidocaine produce short-term analgesia, with intramuscular collagen demonstrating superior early effects to lidocaine [42], though long-term benefits remain uncertain. Despite their analgesic potential, these needling and injectable therapies offer adjunctive, rather than primary, benefit and are best reserved for persistent, function-limiting myofascial pain.

#### 4.1.2 Management of myalgia

Myalgia responds most favorably to conservative, multimodal interventions that combine patient education, behavioral therapy, and physiotherapeutic strategies. Across included RCTs, PE/BT/SC reduced pain intensity, headache frequency, and emotional distress, supporting the role of biopsychosocial interventions for muscular TMD. Manual therapy, Diacutaneous Fibrolysis, and masticatory muscle endurance exercises improved pain-free opening, decreased muscle fatigue, and enhanced functional performance findings consistent with broader literature showing significant gains in muscle efficiency and mobility following orofacial physiotherapy [148]. Stabilization splints, CAD/CAM appliances, and thermoformed splints provided modest improvement in disability and psychosocial parameters, though their additive benefit over behavioral therapy alone was limited, consistent with evidence suggesting greater short-term benefits of stabilization splints while demonstrating generally comparable long-term and superior outcomes across occlusal splint modalities [142, 149]. Physical modalities such as LLLT, PSWT, US consistently reduced pain and improved jaw function, notably [68, 70], HILT showing short and long-term effects in several trials [72]. Consistent with external analyses confirming laser-based therapy as an effective analgesic adjunct [143, 150]. Active Repetitive transcranial magnetic stimulation (rTMS) can lead to significant, yet mild, reductions in pain intensity and pain unpleasantness among individuals with TMD [73]. NSAIDs and muscle relaxants provided short-term relief only [70].

Acupuncture demonstrated significant benefit in relieving pain in patients with TMD and improved unassisted mouth opening, with effects comparable to task-specific splint therapy, reflecting robust neuromodulatory influence [146]. Among the injectable therapies, saline injections provided only transient placebo-like improvements. The analgesic benefit of BoNT/A remained inconsistent across RCTs, with no significant advantage over saline [74], supported by the study that found no better effect than placebo [151]. BoNT/A should not be routinely used for myogenous TMD due to modest efficacy and the risk of muscle weakness [75]. EGF injections showed early promise but remain experimental and didn’t give any advantage, with insufficient evidence for clinical recommendation [76]. Overall, minimally invasive therapies provide meaningful adjunctive benefit but should not replace the rehabilitative foundation essential for muscular recovery.

#### 4.1.3 Management of arthralgia

Arthralgia responds favorably to structured conservative care that combines patient education, behavioral therapy, and multimodal physiotherapy. Across included RCTs, education plus self-care improved psychosocial contributors and normalized mandibular movement patterns, especially in chronic painful TMD [59, 78]. Manual therapy and jog-manipulation techniques consistently reduced joint pain and restored functional range effects supported by broader evidence that craniomandibular manual therapy yields moderate improvements in pain and MMO [14]. Jaw exercises demonstrated additional benefit in increasing mouth opening and reducing functional limitation [28]. Occlusal splints including Michigan stabilization splint, and CAD/CAM splint designs reduced pain-related disability, oxidative stress markers, and depressive symptoms [39, 80]. These findings align with meta-analytic data showing that stabilization splints provide short-term benefit but perform comparably to other conservative modalities over longer follow-up [142]. Adjunct physical modalities such as PBMT, LLLT, US, and TENS offered additional analgesia and modest functional improvement, consistent with evidence that laser-based therapies provide small-to-moderate pain reduction in arthrogenous TMD [143]. Pharmacologic therapies including amitriptyline or tenoxicam supported chronic pain control and sleep quality [90], in contrast, the Glucosamine hydrochloride, chondroitin sulfate, methylsulfonylmethane (GCM) supplementation produced no additional clinical benefit or improvement for the patients [82].

When conservative measures fail to achieve meaningful improvement, minimally invasive interventions, arthrocentesis, intra-articular injections, and needling therapies become appropriate escalation options. Arthrocentesis demonstrated robust pain reduction using saline or Ringer’s lactate, with HA- or PRP-augmented lavage yielding greater long-term improvement in MMO and palpation tenderness [83, 88]. Evidence showed that corticosteroid injections including methylprednisolone, betamethasone, and triamcinolone can reduce pain and improve mouth opening in Temporomandibular Joint (TMJ) osteoarthritis [32, 82, 87, 88], in agreement with international reviews discouraging steroids for degenerative joint inflammation that corticosteroid injection provide favorable clinical improvements in TMJ osteoarthritis patients [152]. Visco supplementation protocols of five sessions outperformed single-session HA in reducing pain over six months, though functional outcomes were similar, suggesting that extended protocols provide analgesic value when long-term pain is the main concern [89]. Among injections, PRP achieved superior pain reduction compared with HA and corticosteroids, consistent with meta-analyses indicating PRP’s regenerative and anti-inflammatory advantages in TMJ osteoarthritis [153, 154, 155]. Acupuncture and dry needling improved joint pain, emotional well-being, and local oxygen saturation, reflecting their neuromodulatory effects demonstrated in broader pain research [145].

#### 4.1.4 Management of disc displacement with reduction

Disc displacement with reduction mainly causes joint clicking due to disrupted disc-condyle coordination. RCTs show structured conservative management is the most effective and least invasive first-line treatment. PE/BT/SC interventions reduce clicking, improve perceived effectiveness, and enhance jaw function, with counselling plus jaw exercises proving more effective than counselling alone [93, 94, 95, 96]. Physiotherapy particularly supervised jaw exercises yielded greater improvements in pain, neck disability, mood, and somatization than home-based exercise programs, reflecting the importance of therapist-guided motor control in disc recapture and muscular relaxation [94, 95, 96]. ARS demonstrated greater improvements in mouth opening and unique suppression of joint clicking compared with self-care alone [93, 97], consistent with external literature showing ARS can temporarily stabilize disc-condyle alignment and effective in reducing pain and popping symptoms compared to physical or behavioral therapy [156]. Conventional occlusal splint also reduced clicking severity and significantly more effective at improving mouth opening [157, 158]. LLLT and US provided additional early-phase analgesia and functional gains, consistent with recent evidence reporting improved pain relief and mandibular function following LLLT in patients with TMD, particularly with higher laser wavelengths [159]. Although propranolol was associated with a 30–50% improvement in pain and enhanced well-being for individuals with chronic myogenous TMD, its effect did not reach statistical significance compared to placebo; therefore, its use is not recommended [101].

Minimally invasive options are reserved for DDR cases that continue to produce painful clicking or functional limitation after optimized conservative care. Injections including 25% dextrose, BoNT/A, and Saline offered variable benefits. Dextrose prolotherapy was effective, easy to administer, and safe, improving pain and maximum interincisal opening (MIO) with high patient satisfaction; however, effects did not consistently exceed arthrocentesis [99, 160]. BoNT/A was shown to reduce clicking and joint pain, with some studies reporting faster symptom relief compared to ARS [97]. External sources also support its effectiveness in relieving pain and significantly decreased muscle activity and biting force [161].

#### 4.1.5 Management of disk displacement without reduction

DDWoR often presents with acute limitation in mouth opening, and conservative therapy remains an essential first-line intervention. PE/BT/SC approaches improved pain, mandibular movement patterns, and functional disability [61, 77, 102, 103]. Jog manipulation added short-term improvement in mouth opening but showed no long-term superiority over conventional exercise-based rehabilitation [61]. ARS, OS, and CAD/CAM splints all reduced pain and improved oral health-related quality of life. However, CAD/CAM splints did not show better clinical outcomes than conventional splints [66], LLLT and TENS reduced pain and improved function, with LLLT demonstrating greater mid-treatment improvement than TENS [110]. Studies confirm that LLLT is a safe, effective, and non-invasive treatment for TMD, providing significant pain relief and functional improvement [162].

DDWoR trials revealed minimally invasive procedures that primarily arthrocentesis and adjunctive injectables provide substantial benefit. Conventional double-puncture, single-puncture, and US-guided techniques provide equivalent reductions in pain and similar improvements in maximal interincisal opening [105, 106, 107], although single-puncture and US-guided methods were faster and required fewer needle manipulations [108]. Both double- puncture and single-puncture arthrocentesis methods are equally effective for patients during the procedure [163]. PRP Lavage consistently produced the greatest long-term improvement in pain, mastication efficiency, and mandibular function, outperforming HA and lavage alone [164]. Findings strongly supported by meta-analytic evidence demonstrating PRP’s regenerative and anti-inflammatory effects in chronic intra-articular pathology [153]. HA injections also significantly improved pain and MMO, though their durability was less than PRP [109].

#### 4.1.6 Management of degenerative joint disease

DJD represents a chronic, inflammatory degenerative process of the TMJ, and conservative therapy remains the essential foundation of care. Across the included RCTs, PE/BT/SC protocols reduced pain intensity, improved function, and optimized mandibular movement patterns, particularly in women with chronic painful TMDs, where education and self-care strategies effectively corrected dysfunctional mechanics [59, 77, 78, 103]. Physical therapy further enhanced mandibular mobility and reduced joint loading [59, 77]. ARS therapy demonstrated potential for condylar repair and remodeling in adolescents and young adults with early DJD [103]. LLLT show superior pain reduction compared with ultrasound and TENS consistent with recent literature [159]. Long-term pulsed radiofrequency improved pain and patient satisfaction [78]. Oral glucosamine hydrochloride provided additional benefit when combined with intra-articular HA, consistent with studies reporting favorable symptomatic and functional outcomes in patients with TMD compared with ibuprofen and paracetamol [165]. The study supports a hybrid biochemical approach, like peripheral joint osteoarthritis management, by confirming glucosamine’s role as a chondroprotective agent [166].

When conservative measures fail to provide adequate relief, minimally invasive interventions particularly arthrocentesis and biologic injectables offer significant benefit. Conventional arthrocentesis effectively reduces joint pain and improves mouth opening by eliminating inflammatory mediators and adhesions within the superior joint space [77]. Adjunct HA injections enhance lubrication and joint biomechanics, with studies demonstrating greater improvements in pain and function compared with arthrocentesis alone [78]. DJD data revealed durable pain relief when arthrocentesis or HA injection was combined with oral glucosamine [111]. Regarding injectables, BoNT/A showed no superiority to saline in chronic muscular TMD and lacks evidence for intra-articular use in DJD [75].

#### 4.1.7 Management of headache attributed to TMD

Headache attributed to TMD frequently coexists with masticatory muscle hyperactivity and central sensitization, making conservative care the essential foundation of treatment. RCTs show that PE/BT/SC programs lower headache frequency, facial pain, and functional limitations, similar to results in general myogenous TMD treatment. Pulsed radiofrequency combined with conventional conservative therapy provided durable pain reduction and patient satisfaction [78], BoNT/A injections provided short-term relief of tender points and headache intensity [112]. But in other study its pain relief compared to saline placebo does not provide extra relief over saline [74].

#### 4.1.8 Management of mixed TMD

Mixed TMD, which are the most frequently encountered clinical presentations, characterized by both muscular and joint symptoms [6]. PE/BT/SC interventions consistently reduced pain intensity, improved sleep quality, and enhanced quality-of-life parameters [113, 114, 115, 116, 117, 118], internet-based behavioral therapy still understudied [113]. Cervical-mandibular manual therapy, Post-Isometric Relaxation (PIR), and structured exercise programs, yielded significant improvements in pain, mandibular range, and psychological outcomes. Findings aligned with broader literature show that addressing cervical mandibular interactions enhances TMD recovery [167]. Manual therapy generally outperformed Bowen’s technique, highlighting the importance of targeted joint–muscle mobilization [121]. Michigan splint, stabilization splint, SOVA, CAD/CAM, and BFB splints shows reduced pain, bruxism activity, muscle tenderness, and sleep disturbances [113, 115, 116, 117, 118, 123, 124], The Michigan splint is a type of stabilization splint, not a separate intervention. Both are hard-acrylic maxillary occlusal appliances designed to reduce parafunctional loading and muscle hyperactivity. The BFB splints demonstrate specific benefits in reducing nocturnal bruxism bursts and improving patient-reported pain [125, 168]. The SOVA splint study found notable differences in sleep bruxism associated with TMD, possibly due to the SOVA’s softer material and larger occlusal contact areas compared to the Michigan splint [126]. LLLT, TENS, ozone therapy, PBMT, and ultrasound further contributed to pain reduction and improved mandibular function, with LLLT offering greater efficacy in muscle tenderness remission [169]. The Bioactive Ozone Therapy shows equal result in reducing pain and increasing MMO [123]. Pharmacologic therapy with duloxetine improved pain and function when used adjunctively but does not impact anxiety, depression, or IL-6 levels [122, 170]. These trials confirm that no single conservative therapy is best for all cases, but combining manual therapy, exercise, behavioral strategies, and splint therapy consistently yields the greatest benefit aligning with meta-analytic evidence that multimodal approaches are most effective for mixed TMD [135].

If symptoms continue after thorough conservative care, minimally invasive procedures may be considered for selected mixed TMD cases. Arthrocentesis reduces joint pain and improves mouth opening, especially when combined with duloxetine, benefiting patients with central sensitization [122]. Needling-based therapies including scalp acupuncture and auricular acupuncture provided short-term pain reductions. But scalp acupuncture is not effective in improving sleep or quality of life [115]. Occlusal equilibration therapy demonstrated clinically significant improvements in facial pain and mouth opening with favorable safety, indicating potential benefit in selected patients with occlusal instability [119, 134].

### 4.2 Clinical implication

The most important part of managing TMD is still conservative care that is based on diagnosis and uses more than one method. Therapeutic integration and patient adherence are more crucial for achieving successful outcomes than any singular treatment. The results highlight the imperative of screening for psychological comorbidities, including anxiety, depression, and catastrophizing, which affect pain persistence and treatment efficacy. Clinicians should begin with PE/BT/SC, progress to behavioral therapy and multimodal physiotherapy, manual therapy and jaw exercises as needed, and consider occlusal splints and adjunct physical modalities for additional pain relief and functional improvement. If pain persists beyond 6–12 weeks its can be considered for using minimally invasive treatment. For Myofascial Pain, Dry needling is recommended due to its consistent impact on trigger-point pain and mouth opening, followed by auricular acupuncture for patients who prefer non-needling modalities. Lidocaine or collagen MD injections may be considered for short-term relief but should not replace comprehensive rehabilitation. Overall, emphasis should remain on multimodal, reversible therapies that address muscular dysfunction, psychosocial contributors, and functional impairments. For Myalgia, dry needling should be considered the preferred minimally invasive option, followed by acupuncture for patients favoring non-trigger-point approaches. BoNT/A is not recommended as a routine treatment due to limited superiority over placebo, and EGF injections remain investigational. For Arthralgia, arthrocentesis with HA is preferred as the minimally invasive first choice due to consistent efficacy, safety, and lower injection frequency. Steroid injections can be considered to use besides the other medicine. PRP may be recommended for refractory cases or suspected early degenerative changes, given its superior long-term analgesia. Acupuncture or dry needling may serve as supportive options but should not replace joint-directed intervention. For DDR, Dextrose prolotherapy may be suitable for patients seeking non-surgical intervention with good safety and functional benefit. BoNT/A may be considered only in selected refractory cases where muscular hyperactivity contributes to persistent clicking. For DDWoR, clinicians should escalate to arthrocentesis, with single-puncture or ultrasound-guided approaches preferred for procedural ease and efficiency. PRP is recommended as the most effective adjunct for long-term pain and function, followed by HA when PRP is unavailable. For DJD, arthrocentesis combined with HA, given its consistent improvement in pain and biomechanics, BoNT/A and saline injections are not recommended due to lack of therapeutic advantage. ARS may be considered only in early DJD with favorable disc–condyle relationships. For Headache attributed to TMD, BoNT/A should only be considered for select refractory cases, given its variable benefit and no clear superiority to saline in trials. Saline or BoNT/A injections are not recommended as first-line or early escalation treatments. Conservative neuromuscular stabilization should be prioritized before minimally invasive procedures. For Mixed TMD, clinicians may consider arthrocentesis and combine duloxetine in cases with centralized or chronic pain features. Acupuncture may be used for short-term analgesia but should not be relied upon as a sole therapy for sleep or psychosocial impairment. Occlusal equilibration can be considered when occlusal disturbances contribute to chronic symptoms. Based on 11 RCTs, several therapeutic interventions are supported for the management of TMD specifically in female patients. These include stabilization splint therapy, BoNT/A injections, and various laser and light therapies such as HILT, LLLT, and PBMT. Physical interventions like kinesio tapping (KT) and mandibular exercise (MME) should also be considered. Treatment efficacy in this population must be rigorously monitored, primarily by assessing pain intensity using scales such as the VAS and NRS, and by evaluating functional capacity through measures like MMO and PPT. In addition, the prioritization of minimally invasive interventions should be guided by the specific TMD subtype. Identifying subtype hierarchies helps clinicians match minimally invasive treatments to the pathology, so escalation from conservative care stays logical and personalised.

### 4.3 Strength, limitation, and future direction

The strength of this review is that it looks at 129 RCTs covering conservative and minimally invasive interventions in a way that separates them by diagnosis. Aligning evidence with diagnostic categories, it establishes clinical pathways for personalized care, enhancing prior reviews of diverse TMD populations. Most studies reported pain intensity using validated scales such as VAS, alongside functional mobility measures such as MMO, enabling consistent comparison of clinical response across diagnostic subtypes. The review also captured outcomes related to psychological and emotional factors, including stress, catastrophizing, and depressive symptoms, as well as quality-of-life indicators, reflecting the biopsychosocial nature of TMD. Importantly, several RCTs incorporated physiological or objective markers including EMG activity, biomarkers such as IL-6, Salivary Oxidative Stress Markers, and Salivary Cortisol indices which provide valuable mechanistic insight into treatment response. This review is the use of the RoB2 framework to assess risk of bias in all included RCTs. The scoping design encompassed various modalities and innovative treatments, providing a thorough overview of the practice.

There are a few limitations that need to be thought about. The variability in diagnostic criteria among studies requires the omission of the effective intervention, treatment dosage, and outcomes from quantitative analysis. The sample sizes were small, and the follow-up periods were rarely longer than six months, which made it hard to see what happened in the long term. However, many primary studies had methodological limitations. While a third of trials were low risk, most showed “some concerns” due to poor reporting of allocation concealment, difficulties blinding participants for certain interventions, and inconsistent adherence to home-exercise protocols potentially affecting treatment effect estimates. Modest sample sizes and limited long-term follow-up further restrict conclusions about sustained efficacy. This review also lacks to population differences, especially sex and region. Over 80% of RCT participants were women, which matches TMD epidemiology but hinders treatment comparisons by sex; women often report higher pain and psychosocial issues, so absence of sex-based analysis limits clinical relevance. Additionally, a quarter of studies were from Brazil, with little evidence from Africa, Southeast Asia, or other low-resource regions, raising concerns about global applicability where minimally invasive treatments may be unavailable.

Future research should prioritize multicenter, randomized studies employing standardized DC/TMD diagnostics and core outcomes for pain, mandibular function, psychosocial distress, and quality of life, accompanied by long-term follow-up (≥12 months) and should include sex-specific data and recruit more diverse populations to improve generalizability. Incorporating psychosocial and biological markers will facilitate the development of precision medicine algorithms to forecast treatment efficacy for subsequent systematic review. Comparative analyses of multimodal treatments versus single-modality care will elucidate synergistic effects. Investigations should explore the mechanisms that connect joint pathology to central pain modulation. Implementation studies of tele-rehabilitation, digital platforms, and cost-effectiveness can enhance the accessibility and adherence to TMD care. Importantly, upcoming RCTs should focus on using robust randomization methods, enhancing blinding whenever possible, and ensuring reports follow Consolidated Standards of Reporting Trials (CONSORT) and RoB2 standards.

## 5. Conclusions

This scoping review demonstrates that the management is most effective when supported by a diagnosis-based framework. An analysis of 129 RCTs shows that conservative treatments, such as patient education, behavior modification, exercise therapy, and occlusal splints, can greatly reduce pain and improve jaw function. Patients with persistent symptoms can benefit from minimally invasive techniques, such as arthrocentesis, needling, and injections. Diagnosis-specific symptoms should be used as escalation rather than replacement strategies for suitable treatment approach. Evidence shows that effective TMD treatment requires personalized, holistic care that considers both physical and psychosocial aspects. Future studies should be focused on standardized diagnostic criteria, longer follow-up, and multidisciplinary strategies to enhance clinical outcomes.

## Supplementary Material

Supplementary material associated with this article can be found, in the online version, at https://files.jofph.com/ files/article/2054073078902603776/attachment/ Supplementary%20material.zip.




