# Supplementary material for: Diagnosis-based pathways for conservative and minimally invasive management of temporomandibular disorders: a scoping review
Source: J Oral Facial Pain Headache. 2026 May 12;40(3):26–51. doi: 10.22514/jofph.2026.034 (PMC13223916; doi:10.22514/jofph.2026.034)
Supplement: Supplementary file 2 [file Supplementary-material-2.docx]

Supplementary material

Supplementary Table 1. Study characteristics.

| No | Author, Year | Country | Study Design | Population, Age, Sex Distribution | Diagnostic Framework |
| --- | --- | --- | --- | --- | --- |
| 1 | Abbasgholizadeh (2020) | Turkey (Istanbul) | RCT | n = 45, Mean age 29.9 ± 9.20 year, 84.4% female, 15.6% Male | RDC/TMD |
| 2 | Aguiar (2023) | Brazil | RCT | n = 148, Mean age 38.05 ± 10.85 year, 80.4% female | RDC/TMD |
| 3 | Aisaiti (2021) | China | RCT | n = 100, Mean age 34.4 ± 12.6 year, 68% female, 32% Male | DC/TMD |
| 4 | Al-Quisi (2023) | Iraq | RCT | n = 60, Mean age 20.6 year, 83% female, 17% Male | DC/TMD |
| 5 | Alajbeg IZ (2020) | Croatia | RCT | n = 34, Mean age 36.1 ± 11.95 year, 100% female | DC/TMD |
| 6 | Aroca (2022) | Brazil | RCT | n = 41 (completed), Mean age 26.63 year, 92.7% female, 7.3% male | RDC/TMD |
| 7 | Babiloni (2024) | Canada | RCT | n = 41, Mean age 26.63 ± 8.57 year, 100% female | DC/TMD |
| 8 | Baker (2015) | Sweden | RCT | n = 45 (initial), n = 34 (responded to follow-up questionnaire), Mean age 38.9 ± 15 year, 91.2% female, 8.8% male | RDC/TMD |
| 9 | Barbosa (2019) | Brazil | RCT | n = 34, Mean age Intervention 30 ± 7 year, Placebo 26 ± 8 year (46 women randomized, 34 completed) | RDC/TMD |
| 10 | Benli (2021) | USA, Turkey, France, Switzerland | RCT | n = 91 (31 LLLT, 30 Placebo, 30 Control), Age range 34–67 year, 93.3% female | DC/TMD |
| 11 | Bergmann (2020) | Germany | RCT | n = 41, Mean age BFB 37.6 ± 11 year, AOS 41.3 ± 14.2 year; BFB 52.6% female, 47.4% male; AOS 55% female, 45% male | RDC/TMD |
| 12 | Bhargava (2019) | India | RCD | n = 20, Above 18 year. Age, gender, and male/female percentages not specified | DC/TMD |
| 13 | Bijelic (2025) | Sweden | RCT | n = 100, Mean age IBT 16.6 year, OAT 16.1 year, 90% female, 10% male | DC/TMD |
| 14 | Brakus (2025) | Croatia | RCT | n = 31, Mean age 45.1 ± 16.8 year (initial n = 40), 90% female (n = 28), 10% male (n = 3) (of completed participants) | RDC/TMD |
| 15 | Brochado (2018) | Brazil | RCT | n = 41, Mean age 44.5 ± 17.1 year, 95.1% female, 4.9% male | RDC/TMD |
| 16 | Celakil (2017) | Turkey | RCT | n = 40, Mean age 33 ± 9.66 year (OG)/34.70 ± 10.13 year (OCSG), 100% female | RDC/TMD |
| 17 | Costa (2015) | Brazil | RCT | n = 41, Group 1 (n = 17): Mean age 36 ± 6.7 year, 90% female | RDC/TMD |
| 18 | Costa (2015) | Brazil | RCT | n = 60, Mean age (Group 1) 36 ± 6.6 year, (Group 2) 27.7 ± 6.7 year, 90% female in both groups | RDC/TMD |
| 19 | Costa (2017) | Brazil | RCT | n = 60, Mean age 38.8 ± 14.2 year, 90% female, 10% male | RDC/TMD |
| 20 | Darwin (2024) | India | RCT | n = 45, Mean age 30.3 ± 9.1 year (Th US), 27.5 ± 6.7 year (TENS), 27.4 ± 6.0 year (LLLT), 53.3% female, 46.7% male | RDC/TMD |
| 21 | De Oliveira Chami (2020) | Brazil | RCT, double-blind, parallel | n = 20 (LG = 10, PG = 10), Mean age 30.1 year (10.9) LG, 23.6 year (4.0) PG, 65% female (13/20) | RDC/TMD |
| 22 | Delgado de la Serna (2019) | Spain, USA | RCT, parallel-group, multicenter | n = 61, Mean age 43.2 ± 11.2 year, 59% female | RDC/TMD |
| 23 | Deregibus (2021) | Italy | RCT, randomized-controlled | n = 40 (13 males, 27 females), Mean age: 47.2 ± 12.8 year, range 22–56 | DC/TMD |
| 24 | Dhanasekaran (2022) | India | RCT | n = 62, Mean age Group I 29.6 ± 5.8 year, Group II 31.5 ± 10.2 year; 56% female, 44% Male | RDC/TMD |
| 25 | Doepel *.* (2017) | Finland, Sweden | RCT | n = 65, Mean age (local pain group) 38 ± 15 year, (widespread pain group) 37 ± 12 year, 89.2% female, 10.8% Male | RDC/TMD |
| 26 | Dunning (2024) | USA | RCT, single-blinded, multi-center | n = 120, Mean age 41.5 ± 12.8 year, 75% female (90/120) | RDC/TMD |
| 27 | Ekici (2022) | Turkey | RCT, double-blind, controlled | n = 76 (randomized into 2 groups of 38), Age 18–70 year | DC/TMD |
| 28 | Ferreira (2017) | Brazil | RCT, randomized placebo-controlled | n = 40 (active = 20, placebo = 20), Mean age 24.6 ± 3.4 year, 75% female (30/40) | RDC/TMD |
| 29 | Folle (2018) | Brazil | RCT, randomized controlled, blinded | n = 26 (Group 1 = 13, Group 2 = 13), Age: Group 1 30.77 (±7.59), Group 2 37.38 (±10.21) | RDC/TMD |
| 30 | Furquim (2023) | Brazil | RCT, no blind, without no-treated group | n = 54 women (total randomized sample), aged 20–57 year (after exclusions); Initial sample 74 women (age 18–60 year), 100% female (74/74) | DC/TMD |
| 31 | García (2023) | Spain | RCT, single-blind, controlled | n = 50 (DN = 25, MT = 25), Mean age: DN 34.56 ± 8.43 year, MT 38.08 ± 9.75 year, 58% female (29/50) | RDC/TMD |
| 32 | Gębska (2023) | Poland | RCT, randomized controlled | n = 82 women (G1, study group), Median age 28.1, (G2, control group n = 104, Median age 29) | DC/TMD |
| 33 | Gębska (2024) | Poland | RCT, single-blinded, parallel | n = 64 women, age 20–45 year (KTG = 32, CG = 32), 100% female (64/64) | DC/TMD |
| 34 | Gerstner (2020) | USA | RCT | n = 61 (SOVA, N = 30; MI, N = 31), Mean age 25.0 ± 3.06 year, 31 Female 30 Male | RDC/TMD |
| 35 | Giannakopoulos (2016) | Germany | RCT | n = 36, Mean age 41.58 ± 16.68 year, 69.4% female | RDC/TMD |
| 36 | Giannakopoulos (2018) | Germany | RCT, randomized clinical trial | n = 45, Mean age 28.2 ± 6.4 year (sensorimotor), 24.7 ± 3.4 year (splint), 100% female (45/45) | RDC/TMD |
| 37 | Gikić (2021) | Croatia | RCT | n = 45 (30 completed), Mean age 36.44 ± 10.51 year, 100% Female, HFP 60%, LFP 40% (OBC score) | DC/TMD |
| 38 | Giro (2016) | Brazil | RCT | n = 42 (after dropouts), Mean age 36.4 ± 8.8 year, 100% female | RDC/TMD |
| 39 | Godoy (2017) | Brazil | RCT | n = 16 (active LLLT = 9, sham = 7), Age 14 to 23 year | RDC/TMD |
| 40 | Grossmann (2017) | Brazil | RCT | n = 26 (1N = 13, 2N = 13), Age 18+, Mean Age (1N) 42.1 (32.8–51.5), (2N) 39 (29.5–48.4) | RDC/TMD |
| 41 | Grossmann (2021) | Brazil | RCT, single-blind | n = 40 (G1 = 20, G2 = 20), Mean age (G1) 35.90 ± 3.00 (G2) 32.55 ± 2.95, 35 Female, 5 Male | RDC/TMD |
| 42 | Haggag (2022) | Egypt | RCT, double-blind | n = 30 (bilateral DDWR, 60 joints), Mean age 23 (Group I 22.7, Group II 23.9), 100% Female | DC/TMD |
| 43 | Hasanoglu Erbasar *.* (2017) | Turkey | RCT | n = 40, NTI-tss group: Mean age 24.6 ± 9.2 year, 80% female, 20% male; Control group: Mean age 32.25 ± 11.97 year, 85% female, 15% male | RDC/TMD |
| 44 | Herpich (2017) | Brazil | RCT, double-blind, placebo-controlled | n = 60 women (15 per group), Age 18–40 year, BMI <25 kg/m^2^, Myofascial pain (Ia) or Myofascial pain with limited opening (Ib) | RDC/TMD |
| 45 | Herpich (2019) | Brazil | RCT, double-blind | n = 30 women (15 active, 15 sham), Mean age (active) 25.44 ± 5.76 year, (sham) 26.55 ± 4.6 year, 100% Female | RDC/TMD |
| 46 | Huttunen (2018) | Finland | RCT | n = 80, Mean age (splint group) 43.2 ± 13.3 year, (control group) 44.0 ± 13.1 year, 77.5% female, 22.5% Male | RDC/TMD |
| 47 | Hwangbo (2023) | Republic of Korea | RCT | n = 41 (20 experimental, 21 control), age 12–65 (mean 31.3), 75.6% female, 24.4% male | DC/TMD |
| 48 | Isacsson (2019) | Sweden | RCT | n = 54, Mean age (Methylprednisolone) 48 ± 18.6 year, 85% female; (Saline) 56 ± 14.7 year, 78% female | DC/TMD |
| 49 | Javed (2024) | Pakistan | RCT | n = 24, Mean age Group 1: 33.33 ± 7.47 year, Group 2: 34.91 ± 7.86 year | DC/TMD |
| 50 | Jo (2021) | Korea | RCT | n = 86 (final analysis: 45 intervention, 41 control), age 20–61 year (mean 37.5 ± 13.8) | DC/TMD |
| 51 | Kahraman (2025) | Turkey | RCT | n = 40, Mean age 24.8 year, 92.5% female | DC/TMD |
| 52 | Kiliç (2016) | Turkey | RCT | n = 24, Control Group: 12 patients (15 joints), Mean age 35.08 ± 14.84 year, 11 female, 1 male; CS Group: 12 patients (17 joints), Mean age 32.58 ± 9.58 year, 10 female, 2 male | DC/TMD |
| 53 | Kiliç (2016) | Turkey | RCT | n = 31 (49 joints), Mean age 30.48 ± 13.04 year; 10 female, 3 male | DC/TMD |
| 54 | Kim (2023) | Republic of Korea | RCT | n = 21, Mean age 33.95 ± 9.70 year, 90.5% female, 9.5% Male | DC/TMD |
| 55 | Kılıç (2021) | Turkey | RCT | n = 26, Mean age 28.35 ± 10.85 year | RDC/TMD |
| 56 | Kopacz (2020) | Poland | RCT | n = 60, 65% female, 35% Male (MLT: 23 women, 7 men; CT: 16 women, 14 men) | DC/TMD |
| 57 | Kutuk (2019) | Turkey | RCT | n = 60 (31 with lateral pain, 43 with posterior pain) | RDC/TMD |
| 58 | Lei (2019) | China | RCT | n = 59 (67 joints), 86.4% females, Mean age 17.95 ± 4.53 year (range 12–30) | DC/TMD |
| 59 | Leite (2020) | Brazil | RCT | n = 34 women, Mean age 25.25 ± 6.43 year, 100% Female | RDC/TMD |
| 60 | Lennartsson (2025) | Sweden | RCT | n = 72 (7 men, 65 women), Mean age 34.2 year, 90.3% Female, 9.7% Male, Chronic pain >6 mon | DC/TMD |
| 61 | Li (2024) | HK, China, UK | RCT | n = 74 (final analysis), Mean age Group 1: 42.46 ± 16.76 year, Group 2: 47.38 ± 16.52 year, Male 13.5% (both groups) | DC/TMD |
| 62 | Lindfors (2020) | Sweden | RCT | n = 97 (20 men, 77 women), Mean age 35 ± 18 year, 79.4% Female, 20.6% Male | RDC/TMD |
| 63 | Liu (2024) | China | RCT | n = 60, Mean age 44.4 ± 16.7 year, 88.3% female, 11.7% male | DC/TMD |
| 64 | Macedo (2020) | Portugal and Spain | RCT | n = 80, Mean age 43.1 ± 17.7 year, 80% female, 20% Male | DC/TMD |
| 65 | Macedo (2023) | Brazil | RCT | n = 32, Mean age 21.53 ± 3.79 year, 88% female, 12% male | DC/TMD |
| 66 | Machado (2016) | Brazil | RCT | n = 82 (76 women, 6 men), Mean age: 30 ± 9.6 year (control), Chronic TMD | RDC/TMD |
| 67 | Madani (2019) | Iran | RCT | n = 45, Mean age 38 ± 15.3 year, 73% Female, 27% Male | RDC/TMD |
| 68 | Magri (2017) | Brazil | RCT | n = 64 women, Mean age 31.7 ± 5.2 year (range 18–40) | RDC/TMD |
| 69 | Magri (2019) | Brazil | RCT | n = 41 women, Mean age 31.7 ± 5.2 year (range 18–40) | RDC/TMD |
| 70 | Majid (2020) | India | RCT | n = 189 (63 per group), Mean age 35 ± 14 year, 116 Female, 73 Male (range 21–49 year) | RDC/TMD |
| 71 | Malekzadeh (2019) | Sweden | RCT | n = 24, Mean age 29.6 ± 13.3 year, 79.2% female, 20.8% Male | DC/TMD |
| 72 | Malekzadeh (2019) | Sweden | RCT | n = 24 (12 per group), Mean age 29.6 ± 13.3 year, 79% Female, 21% Male (range 18–40) | DC/TMD |
| 73 | Maracci (2020) | Brazil | RCT | n = 30 (11 OS, 10 LLLT, 9 placebo), Mean age 33.27 year, 73.3% Female (range 18–60 year) | RDC/TMD |
| 74 | Monaco (2019) | Italy | RCT | n = 50 women, Mean age 36.8 ± 8.5 year (Study 36.41 ± 6.41, Control 37.02 ± 9.15) | RDC/TMD |
| 75 | Monteiro (2020) | Portugal | RCT | n = 42, Mean age 27.4 ± 9.71 year, 76.2% female, 23.8% Male | RDC/TMD |
| 76 | Moraes (2022) | Brazil | RCT | n = 25, 1 patient excluded from SG (n = 13) due to audiometric changes. CG (n = 12) | RDC/TMD |
| 77 | Nadershah (2019) | Saudi Arabia and Egypt | RCT | n = 202, Mean age 33.8 ± 10.6 year, 54% Female, 46% Male | DC/TMD |
| 78 | Nagata (2015) | Japan | RCT | n = 181 (63 Male, 118 Female), Mean age 42.1 ± 18.25 year (NS 43.1 ± 17.6, NS + S 41.1 ± 18.9) | RDC/TMD |
| 79 | Nagata (2019) | Japan | RCT | n = 61 (11 men, 50 women, mean age 49.6 ± 25 year with mouth opening limitation ≤35 mm) | DC/TMD |
| 80 | Nahar (2023) | India | RCT | n = 60 (30 per group), Patients >16 year, 44 Female, 16 Male | RDC/TMD |
| 81 | Nardini (2015) | Italy | RCT | n = 30 (10 per group), 9+ Female per group, Mean age 56.8 ± 2.7 to 60.1 ± 4.5 year | RDC/TMD |
| 82 | Nemani (2024) | India | RCT | n = 48 (12 per group), Both genders (range 18–60), diagnosed with chronic cervical and TMJ pain | RDC/TMD |
| 83 | Nitecka-Buchta (2018) | Poland | RCT | n = 43, Mean age 39.97 ± 3.78 year, 60.47% female, 39.53% Male | DC/TMD |
| 84 | Nordenflycht (2022) | Chile | RCT | n = 46 (10 Male, 34 Female), Mean age 24.02 year, 77.27% Female (range 18–40 year old) | DC/TMD |
| 85 | Nordenflycht (2024) | Chile | RCT | n = 48 (35 Female, 13 Male), Mean age SM group 27.6 ± 5.9 year, SM + EX group 24.3 ± 4.2 year (18–40) | DC/TMD |
| 86 | Özden (2018) | Turkey | RCT | n = 40 (20 SDN, 20 DDN), 24 Female, 16 Male (range 18–65), with trigger points in masseter | RDC/TMD |
| 87 | Packer (2015) | Brazil | RCT | n = 32 women, Mean age 24.78 ± 5.41 year (range 18–40), all diagnosed with TMD (Ia or Ib) | RDC/TMD |
| 88 | Patil and Aileni (2017) | Saudi Arabia | RCT | n = 36, Mean age TENS group 32.91 ± 12.57 year, HE group 34 ± 7.4 year. TENS group: 61.11% female, 38.88% male. HE group: 66.66% female, 33.33% male | RDC/TMD |
| 89 | Patil DJ, Dheer DS (2023) | India | RCT | n = 40, Mean age 25.33 ± 3.20 year, 80% female, 20% Male | DC/TMD |
| 90 | Peixoto (2021) | Brazil | RCT | n = 60, (convenience sample), 81.7% mixed TMD, Mean age not reported (18–65) | RDC/TMD |
| 91 | Percin (2025) | Turkey | RCT | n = 50, Mean age 20.5 year, 100% female | DC/TMD |
| 92 | Perez (2015) | Spain | RCT | n = 48 (24 DDN, 24 drug), Mean age 34.3 ± 13.8 year (DDN), 35.5 ± 11.2 year (drug) (range 18–65) | RDC/TMD |
| 93 | Pho Duc (2016) | Germany | RCT | n = 32 (68.75% women, 28.51 ± 7.13 year old), 16 per group (range 20–50 year old) | RDC/TMD |
| 94 | Pihut (2018) | Poland | RCT | n = 112 (83 Female, 29 Male), Mean age 31 year (range 24–45) | DC/TMD |
| 95 | Polat (2021) | Turkey | RCT | n = 59 (46 Female, 13 Male), Mean age E = 20.45 ± 1.50, LLLT = 21.41 ± 4.3, MPR = 20.95 ± 1.83 (range 18–30) | RDC/TMD |
| 96 | Prati (2024) | Italy | RCT | n = 48 (16 per group), Mean age Conventional Splint 27.06 ± 10.60, Modified Occlusal Splint 22.25 ± 10.65, Remodeling Exercise 26.63 ± 11.39 (range 18+) | DC/TMD |
| 97 | Qataya (2025) | Egypt | RCT | n = 29 (25 Female, 4 Male), Mean age 27.74 year (range 20–50), chronic painful TMD | DC/TMD |
| 98 | Rady (2022) | Egypt | RCT | n = 27 (9 per group), Mean age 23.22–24.22 ± 2.1–2.9, 88.9–100% Female (range 20–40) | DC/TMD |
| 99 | Ram (2021) | India | RCT | n = 160 (73 Male, 87 Female), Mean age 39.44 ± 10.34 (Group A 37.60 ± 10.55, Group B 42.25 ± 9.85, Group C 40.42 ± 10.41, Group D 37.48 ± 10.15), Age >20 year | DC/TMD |
| 100 | Resende (2019) | Brazil | RCT, blind, controlled | n = 89 (after withdrawals/death), Mean age 28 ± 9.34 year, 80.9% female (72/89) | RDC/TMD |
| 101 | Rezaie (2022) | Iran | RCT | n = 30, Mean age 28.33 ± 5.43 (control), 27.65 ± 4.04 (intervention), 60% female (control), 53.3% female (intervention) | DC/TMD |
| 102 | Rezazadeh (2022) | Iran | RCT | n = 36, Mean age 26.53 year (BoNT/A: 28.78, Placebo: 24.78), 19 female, 17 male | RDC/TMD |
| 103 | Ritto (2022) | Brazil | RCT | n = 59 (60 selected, 1 excluded), Mean age 34.17 ± 13.1 year, 88.1% female, 11.9% male | DC/TMD |
| 104 | Rodríguez (2024) | Mexico | RCT | n = 91, Mean age 31.2, 72% female, 28% Male | DC/TMD |
| 105 | Rodriguez-Blanco (2015) | Spain | RCT | n = 60 (41 female, 19 male), Mean age 35 ± 11.22 year (range 18–50) | RDC/TMD |
| 106 | Saglam (2024) | Turkey | RCT | n = 48 patients, 16 healthy, Mean age 26.56 ± 4.68 to 30.13 ± 8.37 (range 18–30), 81.3% Female, 18.8% Male | DC/TMD |
| 107 | Saini (2025) | India | RCT | n = 80, Mean age 39.08 year, 81% female, 18.75% Male | DC/TMD |
| 108 | Sajedi (2022) | Iran | RCT | n = 60 (34 Female, 26 Male), Mean age 33.88 ± 6.03 year (range 19–48), MPDS patients | RDC/TMD |
| 109 | Sancakli (2015) | Turkey | RCT | n = 30 (21 women, 9 men), Mean age 39.2 ± 2.8 (range 18–60), TMD of muscular origin | RDC/TMD |
| 110 | Santana-Penín (2023) | Spain | RCT | n = 77, Mean age: ET group 29 (IQR 22–38), Sham group 30 (IQR 25–40). ET group: 92.3% female, 7.7% male; Sham group: 92.1% female, 7.9% male | DC/TMD |
| 111 | Şen (2020) | Germany | RCT | n = 41 (38 female), Mean age 40.17 ± 16.61 (Specific 41.56 ± 17.1, Non-specific 39.09 ± 16.52) | DC/TMD |
| 112 | Silva (2024) | Brazil | RCT | n = 24 (79.2% Female), Mean age 32.83 ± 13.45 year (range 18–64), with parafunctional habits | DC/TMD |
| 113 | Simões (2023) | Brazil | RCT | n = 68 (34 per group), Mean age 25.88 ± 7.26 year, 68.4% female | RDC/TMD |
| 114 | Singh (2017) | India | RCT | n = 20 patients, (no age/gender breakdown) | RDC/TMD |
| 115 | Siqueira (2025) | Brazil | RCT | n = 99, Mean age 31.54 ± 10.39 year, 80% Female, 19% Male | DC/TMD |
| 116 | Sitnikova (2024) | Finland | RCT | n = 57, Mean age 38.2 ± 10.4 year, 82.5% female, 17.5% Male | DC/TMD |
| 117 | Sousa (2025) | Portugal, Spain | RCT | n = 60, 80% Female, Mean age Dry Needling 36.8 ± 15.88 | DC/TMD |
| 118 | Souza (2024) | Brazil | RCT | n = 54 women, Mean age not reported (range 18–45), 94.4% joint pain, 77.8% neck pain | RDC/TMD |
| 119 | Taleb (2025) | Syearia | RCT | n = 20 female patients, Mean age not reported (range 18–35) | DC/TMD |
| 120 | Tchivileva (2020) | USA | RCT | n = 199 (ITT), Mean age 34.1 ± 12.7, 77.9% female, 22.1% Male | DC/TMD |
| 121 | Toameh (2019) | Syearia | RCT | n = 30 (6 male, 24 female), Mean age 38.87 ± 6.40 year | DC/TMD |
| 122 | van Grootel (2017) | Scotland | RCT | n = 72 (37 for physiotherapy, 35 for splint therapy), Mean age 30.2 ± 9.6, 93% female, 7% male | RDC/TMD |
| 123 | Wahlund (2017) | Sweden | RCT | n = 167 adolescents, Mean age 15.5 (2.1), 81.4% Girls, 18.6% Boys (range 12–19 year) | RDC/TMD |
| 124 | Wahlund (2021) | Sweden | RCT | n = 83 adolescents, 83% Girls, Mean age 15.6 (2.1) | RDC/TMD |
| 125 | Wänman (2019) | Sweden | RCT | n = 90, Mean age 39.2 ± 15.2, 70% Female, 30% Male (range 18–70 year) | RDC/TMD |
| 126 | Yang (2018) | China | RCT | n = 144, Mean age Group A: 40.1 ± 15.8, Group B: 36.2 ± 15.8, 83.3% female, 16.7% male | RDC/TMD |
| 127 | Yeladandi (2024) | India | RCT | n = 30 (11 males, 19 females), Mean age 33.6 year (range 18–65), clinically diagnosed with TMD | RDC/TMD |
| 128 | Zotelli (2017) | Brazil | RCT | n = 40, Mean age 36.5 ± 8.6, 80% female, 20% Male | RDC/TMD |
| 129 | Pihut (2020) | Poland | RCT | n = 100 (50 per group), Mean age 35.2 year, 78% Female, 22% Male (range 21–43 year) | RDC/TMD |

BFB: biofeedback splint; AOS: anterior open splint; IBT: internet-based therapy; OAT: occlusal appliance therapy; OG: occlusal guard; OCSG: occlusal splint–guided therapy; Th US: therapeutic ultrasound; TENS: transcutaneous electrical nerve stimulation; LLLT: low-level laser therapy; LG: laser group; PG: placebo group; DN: dry needling; MT: manual therapy; KTG: kinesio taping group; CG: control group; SOVA: sleep-optimized variable appliance; MI: minimally invasive; HFP: high-frequency parafunction; LFP: low-frequency parafunction; OBC: occlusal bite control; DDWR: disc displacement with reduction; NTI-tss: nociceptive trigeminal inhibition tension suppression system; BMI: body mass index; MLT: magnetic laser therapy; CT: cryotherapy; OS: occlusal splint; SG: study group; NS: normal saline; NS + S: normal saline plus splint; TMJ: temporomandibular joint; SM: self-management; EX: exercise; SDN: superficial dry needling; DDN: deep dry needling; HE: home exercise; BoNT/A: botulinum toxin type A; MPDS: myofascial pain dysfunction syndrome; IQR: interquartile range; ITT: intention-to-treat; RCT: randomized controlled trial; RDC/TMD: Research Diagnostic Criteria for Temporomandibular Disorders; DC/TMD: Diagnostic Criteria for Temporomandibular Disorders.

Supplementary Table 2. Mapped data.

| Mapped Diagnosis | Mapped Treatment | Author Year | Group A (Control) | Intervention Group B (Treatment) | Conclusion |
| --- | --- | --- | --- | --- | --- |
| Arthralgia | Arthrocentesis/Joint Lavage, Injections | Kiliç (2016) | Arthrocentesis (100 mL Ringer’s lactate) | Arthrocentesis (100 mL Ringer’s lactate) + MPA injection (1 mL, single dose) | Arthrocentesis alone and arthrocentesis with corticosteroid injection resulted in favorable clinical improvements for TMJ OA patients, with no superior outcomes for the combined therapy in terms of range of motion and clinical symptoms. |
|  | Arthrocentesis/Joint Lavage, Injections | Kiliç (2016) | Arthrocentesis + HA injection (1 mL Hyalgan, single session) | Arthrocentesis + PRP injection (1 mL PRP, initial injection + 4 consecutive monthly injections without arthrocentesis) | Arthrocentesis combined with PRP injections was not superior to arthrocentesis combined with a single HA injection for TMJ OA treatment, suggesting HA may be a more acceptable first-line treatment due to fewer injections. |
|  | Arthrocentesis/Joint Lavage, Injections, Pharmacotherapy | Kılıç (2021) | Arthrocentesis + Intraarticular HA injection (single session of Hyalgan, 20 mg/2 mL) | Arthrocentesis + Intraarticular HA injection (single session of Hyalgan, 20 mg/2 mL) followed by 3 months of oral GCM supplementation (750 mg glucosamine hydrochloride, 600 mg chondroitin sulfate, 350 mg methylsulfonylmethane, 2 × 1 dosage daily) | The addition of GCM supplementation following arthrocentesis and intraarticular HA injection did not provide additional clinical benefits for patients with TMJ osteoarthritis compared to arthrocentesis plus HA injection alone. |
|  | Injections | Kutuk (2019) | - PRP injection (1 mL, 1-month interval, 3 months) | - HA injection (1 mL, 1-month interval, 3 months)  - CS injection (1 mL triamcinolone asetate, 1-month interval, 3 months) | Intra-articular PRP injections were more effective in decreasing TMJ palpation pain compared to HA and CS injections in patients with TMJ osteoarthritis. |
|  | Arthrocentesis/Joint Lavage, Oral Splints | Li (2024) | OS only (flat-plane hard acrylic splint for upper arch, nightly use) | Early Arthrocentesis (2-needle, local anaesthesia, 100 mL normal saline lavage) + OS (flat-plane hard acrylic splint for upper arch, nightly use) | Early arthrocentesis combined with OS use is superior to OS use alone for treating TMJ arthralgia, particularly in pain reduction. |
|  | Needling | Liu (2024) | Sham acupuncture (noninvasive Park Sham device at same acupoints) | Acupuncture (bilateral LI4, GB34; affected-side SI19, ST6, ST7; 30-min sessions, 3× weekly for 4 weeks) | Acupuncture is more effective than sham acupuncture in reducing pain, improving jaw function, and improving emotional well-being and sleep qualitywith effects lasting up to 8 weeks. |
|  | Oral Splints, Injections | Macedo (2020) | Bite splint (night use) | Bite splint + Betamethasone injections (1 mL)  Bite splint + Sodium hyaluronate injections (1 mL)  Bite splint + PRP injections (2 mL) | All treatments significantly reduced pain and increased pain-free mouth opening, with the bite splint combined with PRP injection achieving the best long-term success. |
|  | Needling | Macedo (2023) | Sham (no active procedure) | DN (0.20 × 13 mm acupuncture needle, inserted 10 times outward and inward at ~1 movement/s) | DN significantly increased tissue oxygen saturation in the masseter muscle |
|  | Arthrocentesis/Joint Lavage | Nardini (2015) | Single-session Arthrocentesis (medium-molecular weight viscosupplementation HA) | - Single-session (high-molecular weight HA: 7000 kDa, 1 mL)- Five-session (medium-molecular weight HA: 1200 kDa, 1 mL, 5 weekly sessions) | The five-session viscosupplementation protocol is superior to single-session protocols in reducing pain levels for TMJ degenerative disorders over six months, but all three protocols showed similar improvements in most other functional outcomes, including chewing efficiency, functional limitation, perceived efficacy, and mouth opening, suggesting a potential for reducing intervention frequency in future strategies while prioritizing pain management. |
|  | Arthrocentesis/Joint Lavage, PT & EX, Pharmacotherapy, “Patient Education, Behavioral Therapy & Self-Care” | Ritto (2022) | LA injection, soft diet, physiotherapy, CBT | Medication Group: LA injection, medication (tenoxicam) for 30 days, soft diet, physiotherapy, CBT  ALG: Arthrocentesis, soft diet, physiotherapy, CBT  ALMG: Arthrocentesis, medication (tenoxicam 20-mg pills) for 30 days, soft diet, physiotherapy, CBT | Both arthrocentesis and conservative modalities effectively reduced joint pain and increased mandibular opening. |
|  | “Patient Education, Behavioral Therapy & Self-Care”, PT & EX | Giro (2016) | Kinesiographic assessment at T0, T1 (30 days) and T2 (60 days), with education and self-care instructions provided at T1 and reviewed at T2 | EG: Education instructions at T0, followed by education and self-care instructions at T1, and review at T2.  ESG: Education and self-care instructions at T0, with review at T1 and T2. Education included information about TMD, structures involved, causes, prognosis, and self-care for mandibular muscles. Self-care therapies included thermotherapy (moist heat pads, 15 min, 3×/day), mandibular exercises (controlled movements, stretching, resistance, coordination, 6 repetitions, 3×/day), and self-massage (masseter and temporal muscles, 3×/day). | ESG group positively influenced the mandibular movement pattern of women with chronic painful TMDs in the short-term. |
|  | Physical Modalities | Aisaiti (2021) | Placeboo Laser treatment | PBMT therapy (GaAlAs laser, 810 nm, 6 J/cm^2^, 7 sessions, once daily) | Both PBMT and placebo significantly improved TMD pain and jaw function over time. |
|  | Physical Modalities | Babiloni (2024) | Sham rTMS | Active rTMS (30 trains of 50 stimuli, 20 Hz, 80% RMT, 2.5 s train duration, 30 s intertrain interval, 1 session, ~20 min total) | A single session of active rTMS provided immediate, mild analgesic effects for TMD pain and unpleasantness compared to sham. |
|  | Pharmacotherapy | Brakus (2025) | Placeboo | Amitriptyline (25 mg, 2 mon) | Low-dose amitriptyline is an effective treatment for chronic TMD, significantly reducing pain and improving OHRQoL compared to placebo. |
|  | PT & EX, Physical Modalities | Brochado (2018) | Manual Therapy (MT) (21 minutes/session on masticatory muscles and TMJ, 12 sessions over 4 weeks) | PBMT (PBM) (GaAlAs diode laser, 808 nm, 100 mW, 133 J/cm^2^, 4 J/point, 12 sessions over 4 weeks) | PBM, manual therapy, and their combination effectively reduced pain, improved mandibular function, and alleviated negative psychosocial aspects and anxiety in TMD patients. |
|  | Physical Modalities | Costa (2017) | Placebo | PBMT therapy (830 nm infrared laser, 100 mW, 100 J/cm^2^, 2.8 J per point, 5 points bilaterally on temporal and masseter muscles, single session) | PBMT therapy (830 nm) effectively reduced masticatory muscle pain as assessed by palpation but did not significantly impact mouth opening range, indicating its potential as a coadjuvant treatment for TMD-related pain. |
|  | Oral Splints, PT & EX | Gikić (2021) | Control therapy (CT): thin thermoforming foil (0.5 mm, upper jaw, all contacts interfering with simultaneous contact at maximum intercuspation removed, nightly use, 6 months) | - PT: twice a day, 10–20 repetitions each exercise, 6 months).  - Stabilization splint: hard acrylic (1.5 mm thick, upper jaw, centric relation, canine guidance, smooth flat surface; nightly use, 6 months) | While no treatment was superior for spontaneous pain relief, PT was more effective for characteristic pain intensity; in patients with high-frequency parafunction, anxiety, stress, and functional limitation decreased regardless of the applied therapy, suggesting a possible placebo effect. |
|  | Injections | Isacsson (2019) | Saline IA injection (1 mL) | Methylprednisolone IA injection (40 mg/mL, 1 mL) | A single-dose intra-articular injection of methylprednisolone did not provide additional pain relief compared to saline in patients with TMJ arthralgia. |
|  | “Patient Education, Behavioral Therapy & Self-Care”, Physical Modalities | Jo (2021) | Sham pulsed radiofrequency + conventional noninvasive TMD treatment (counselling for behavioral therapy, occlusal stabilization splint, thermal moist heat, US, low-level laser, transcutaneous electrical nerve stimulation, aceclofenac 100 mg bid for 14 days, cyclobenzaprine 10 mg qhs for 14 days) | Pulsed radiofrequency (1.5 W, 1 MHz, 10 min/session, once a week for 12 weeks, on both masticatory muscles) + conventional noninvasive TMD treatment (counselling for behavioral therapy, occlusal stabilization splint, thermal moist heat, US, low-level laser, transcutaneous electrical nerve stimulation, aceclofenac 100 mg bid for 14 days, cyclobenzaprine 10 mg qhs for 14 days) | Long-term regular pulsed radiofrequency therapy, combined with conventional treatments, effectively and durably reduced TMD pain and improved patient satisfaction with minimal discomfort. |
|  | Oral Splints | Alajbeg IZ (2020) | Placebo splint (0.5 mm thin transparent foil, night-time use, 6 months) | Stabilization splint (full-coverage hard acrylic upper appliance, 1.5 mm thick, night-time use, 6 months) | Stabilization splint reduced pain-related disability, depressive symptoms, and oxidative stress markers. |
|  | Oral Splints, “Patient Education, Behavioral Therapy & Self-Care” | Giannakopoulos (2016) | Counselling only (waiting list for Michigan-type hard splint after 2 weeks) | Group A: Prefabricated oral splints (Aqualizer) worn during sleep and for at least 6 h/day (2 weeks)  Group B: Individualized vacuum-formed co-polyester oral splints worn during sleep and for at least 6 h/day (2 weeks) | Counselling combined with a vacuum-formed splint appears to be a favorable immediate short-term option for painful TMD symptoms. |
|  | Injections, Arthrocentesis/Joint Lavage | Baker (2015) | Local anaesthetics | Local anaesthetics and lavage (AL) (50 mL saline) | Local anaesthetics and lavage demonstrated equivalent effectiveness to local anaesthetics alone in reducing pain, improving physical and emotional functioning, and overall global improvement in painful TMJ DDwoR patients over a 3-year period. |
|  | Physical Modalities | Yeladandi (2024) | TENS therapy | LLLT (980 nm, 1 Joule, 500 mW, 6 minutes, 8 sessions (2/week for 4 weeks)), US therapy (1.0 W/cm^2^, pulsed mode, 1 MHz, 10 minutes, 12 sessions (1/day for 2 weeks)) | Both LLLT and US therapy are more effective than TENS in reducing TMD pain post-treatment, with LLLT showing superiority in pain reduction at mid-treatment; all three therapies are equally effective in improving mouth opening post-treatment, while LLLT and US show better mid-treatment mouth opening than TENS. |
|  | Injections | Sitnikova (2024) | SS injections | BoNT/A injections (50 U, single dose, bilateral into temporal and masseter muscles) | A 50-unit injection of BoNT/A might help with muscle pain symptoms, but the specific effect of the drug on pain compared to the placebo is not obvious. |
|  | Physical Modalities | Madani (2019) | Sham laser (5 weeks, 2×/week, 10 sessions) | LLLT (GaAlAs laser, 810 nm, 200 mW, 30 s/point, 21 J/cm^2^, 5 weeks, 2×/week, 10 sessions) - LAT (GaAlAs laser, 810 nm, 200 mW, 30 s/point, 21 J/cm^2^, on ST6, ST7, LI4, 5 weeks, 2×/week, 10 sessions) | Both LLLT and LAT are effective in reducing pain and improving excursive and protrusive mandibular movements in TMD patients, with LAT offering a suitable alternative due to shorter chair time, though LLLT showed somewhat better results for TMJ pain and masseter insertion pain. |
|  | PT & EX, “Patient Education, Behavioral Therapy & Self-Care” | Nagata (2019) | Conventional treatment (self-exercise: molar pulldown type + S-MFT, CBT, education for TMD) | Conventional treatment + Jog-manipulation (4 types of manipulations with gauze pivot on last molar, repeated 3× if opening <40 mm, at first visit and subsequent visits until opening <40 mm) | The addition of jog-type mandibular manipulation to comprehensive conventional therapy, including improved therapeutic exercises and CBT, offers a short-term advantage in improving mouth-opening limitation, particularly after the initial treatment, but does not provide long-term superiority over conventional therapy alone for overall TMD symptoms, suggesting that well-executed self-exercises can achieve similar effects over time. |
|  | Injections | Lennartsson (2025) | Saline (2 injections, 3 months apart) | BoNT/A (100U, 2 injections, 3 months apart) | BoNT/A did not offer additional pain relief compared to saline for chronic orofacial myalgia in patients already receiving conservative treatment. |
|  | Oral Splints | Pho Duc (2016) | Conventional stabilization splint | CAD/CAM stabilization splint | Both CAD/CAM and conventional stabilization splints are equally efficacious in reducing various TMD symptoms, including pain, tension, and jaw joint noise, over a 9-month period, but neither significantly improves mandibular movements or condylar asymmetry, suggesting that the advanced manufacturing of CAD/CAM splints does not translate to superior clinical outcomes compared to conventional splints, although it may alleviate a wider range of symptoms. |
|  | “Patient Education, Behavioral Therapy & Self-Care” | Hwangbo (2023) | Conventional treatment (behavioral therapy, drug treatment, physical therapy) | Conventional treatment (behavioral therapy, drug treatment, physical therapy) + Smartphone application for CBT (5–10 min/day, daily use, for 5.9 weeks) | Repetitive CBT via a smartphone application, combined with conventional treatment, improved TMD symptoms, reducing pain and increasing mouth opening, suggesting its potential as a digital therapeutic. |
|  | Oral Splints, PT & EX | Lindfors (2020) | -No treatment (waiting list, 3 months)  -Stabilization appliance (hard acrylic, night use, 3 months) | - JEs (relaxation, free movements, resisted movements, stretching; 3×/day) | JEs effectively reduce pain, headache, and analgesic consumption in masticatory myofascial pain patients and are more cost-effective than stabilization appliances, despite similar pain reduction efficacy between the two active treatments. |
|  | “Patient Education, Behavioral Therapy & Self-Care”, PT & EX | Giro (2016) | Kinesiographic assessment at T0, T1 (30 days) and T2 (60 days), with education and self-care instructions provided at T1 and reviewed at T2 | EG: Education instructions at T0, followed by education and self-care instructions at T1, and review at T2.  ESG: Education and self-care instructions at T0, with review at T1 and T2. Education included information about TMD, structures involved, causes, prognosis, and self-care for mandibular muscles. Self-care therapies included thermotherapy (moist heat pads, 15 min, 3×/day), mandibular exercises (controlled movements, stretching, resistance, coordination, 6 repetitions, 3×/day), and self-massage (masseter and temporal muscles, 3×/day) | ESG group positively influenced the mandibular movement pattern of women with chronic painful TMDs in the short-term. |
|  | Physical Modalities | Aisaiti (2021) | Placeboo Laser treatment | PBMT therapy (GaAlAs laser, 810 nm, 6 J/cm^2^, 7 sessions, once daily) | Both PBMT and placebo significantly improved TMD pain and jaw function over time. |
|  | Physical Modalities | Babiloni (2024) | Sham rTMS | Active rTMS (30 trains of 50 stimuli, 20 Hz, 80% RMT, 2.5 s train duration, 30 s intertrain interval, 1 session, ~20 min total) | A single session of active rTMS provided immediate, mild analgesic effects for TMD pain and unpleasantness compared to sham. |
|  | Pharmacotherapy | Brakus 2025 | Placeboo | Amitriptyline (25 mg, 2 months) | Low-dose amitriptyline is an effective treatment for chronic TMD, significantly reducing pain and improving OHRQoL compared to placebo. |
|  | PT & EX, Physical Modalities | Brochado 2018 | MT (MT) (21 minutes/session on masticatory muscles and TMJ, 12 sessions over 4 weeks) | PBMT (PBM) (GaAlAs diode laser, 808 nm, 100 mW, 133 J/cm², 4 J/point, 12 sessions over 4 weeks) | PBM, manual therapy, and their combination effectively reduced pain, improved mandibular function, and alleviated negative psychosocial aspects and anxiety in TMD patients. |
|  | Physical Modalities | Costa (2017) | Placebo | PBMT therapy (830 nm infrared laser, 100 mW, 100 J/cm², 2.8 J per point, 5 points bilaterally on temporal and masseter muscles, single session) | PBMT therapy (830 nm) effectively reduced masticatory muscle pain as assessed by palpation but did not significantly impact mouth opening range, indicating its potential as a coadjuvant treatment for TMD-related pain. |
|  | Oral Splints, PT & EX | Gikić (2021) | Control therapy (CT): thin thermoforming foil (0.5 mm, upper jaw, all contacts interfering with simultaneous contact at maximum intercuspation removed, nightly use, 6 months) | - PT: twice a day, 10–20 repetitions each exercise, 6 months)  - Stabilization splint: hard acrylic (1.5 mm thick, upper jaw, centric relation, canine guidance, smooth flat surface; nightly use, 6 months) | While no treatment was superior for spontaneous pain relief, PT was more effective for characteristic pain intensity; in patients with high-frequency parafunction, anxiety, stress, and functional limitation decreased regardless of the applied therapy, suggesting a possible placebo effect. |
|  | Injections | Isacsson (2019) | Saline IA injection (1 mL) | Methylprednisolone IA injection (40 mg/mL, 1 mL) | A single-dose intra-articular injection of methylprednisolone did not provide additional pain relief compared to saline in patients with TMJ arthralgia. |
|  | “Patient Education, Behavioral Therapy & Self-Care”, Physical Modalities | Jo (2021) | Sham pulsed radiofrequency + conventional noninvasive TMD treatment (counselling for behavioral therapy, occlusal stabilization splint, thermal moist heat, US, low-level laser, transcutaneous electrical nerve stimulation, aceclofenac 100 mg bid for 14 days, cyclobenzaprine 10 mg qhs for 14 days) | Pulsed radiofrequency (1.5 W, 1 MHz, 10 min/session, once a week for 12 weeks, on both masticatory muscles) + conventional noninvasive TMD treatment (counselling for behavioral therapy, occlusal stabilization splint, thermal moist heat, US, low-level laser, transcutaneous electrical nerve stimulation, aceclofenac 100 mg bid for 14 days, cyclobenzaprine 10 mg qhs for 14 days) | Long-term regular pulsed radiofrequency therapy, combined with conventional treatments, effectively and durably reduced TMD pain and improved patient satisfaction with minimal discomfort. |
| Myalgia | Injections | Sitnikova (2024) | SS injections | BoNT/A injections (50 U, single dose, bilateral into temporal and masseter muscles) | A 50-unit injection of BoNT/A might help with muscle pain symptoms, but the specific effect of the drug on pain compared to the placebo is not obvious. |
|  | PT & EX | Barbosa 2019 | Placebo (simulated LLLT) | Masticatory muscle endurance exercises (biting endurance exercises controlled by BFB, 2 times/week, 8 weeks, 20–50% MVIC) | An 8-week protocol of local masticatory muscle endurance exercises effectively reduced oro-facial pain and improved resistance to fatigue and muscle efficiency in women with TMD. |
|  | Physical Modalities | Benli (2021) | Placebo & no treatment | LLLT (GaAlAs semiconductor diode, 808 nm, 70 J/cm², 1.9 J total, 100 mW, 19 s/point, 2 sessions/week for 1 month); Placebo laser (660 nm) | LLLT effectively provided short-term improvements in chewing performance and reduced pain for SLE patients experiencing myogenic TMD. |
|  | Pharmacotherapy, Physical Modalities | Dhanasekaran (2022) | Pharmacotherapy (muscle relaxant & NSAID) | PSWT (ActiPatch, 60 min/day, 5 days) | PSWT significantly decreased pain without adverse effects and showed better and more sustained pain alleviation than pharmacotherapy for symptomatic TMD. |
|  | Oral Splints | Doepel (2017) | Prefabricated oral appliance | Oral appliance (stabilization splint) (night use, 1 year) | Oral appliances improved outcomes regardless of localized or widespread pain. |
|  | Physical Modalities | Leite (2020) | Sham DF (4 weeks, 2×/week) | DF (stainless-steel hook, 4 weeks, 2×/week, 10 min/session) | DF is effective in reducing myalgia, increasing mouth opening, and decreasing functional severity in women with TMD. |
|  | “Patient Education, Behavioral Therapy & Self-Care”, Oral Splints | Nordenflycht (2022) | Behavioral and self-care therapy | - Thermoformed Tough-elastic splint (2mm PET) + BST (used during sleep)  - Thermoformed Soft-elastic splint (3mm EVA) + BST (used during sleep)  - Non-occlusive splint (2mm rigid PET) + BST (used during sleep) | The addition of thermoformed OSs (tough-elastic, soft-elastic, or non-occlusive) to behavioral and self-care therapy does not provide additional benefits for reducing masticatory muscle pain or increasing mandibular range of motion, although tough-elastic splints may offer superior improvement in jaw functional limitation. |
|  | “Patient Education, Behavioral Therapy & Self-Care”, PT & EX | Nordenflycht (2024) | SM | SM + mandibular HEs (EX) (self-massage, self-stretching, strengthening) | The addition of mandibular HEs to a structured SM program does not provide a significant additional impact on masticatory muscle pain, jaw opening range of motion, or functional limitation in the short-term. |
|  | Physical Modalities, Injections | Qataya (2025) | Not applicable | - PLLT (1064 nm Nd-YAG laser, 4 sessions once/week)  - Intramuscular EGF injection (0.1 mL per point, 3 points per muscle, 2 sessions at baseline and day 14) | Both PLLT and EGF injection effectively alleviate pain, improve pain-free opening, and enhance QoL in myogenic TMD patients, with PLLT also significantly improving MMO and demonstrating better longevity for pain relief and sustained pain-free opening compared to EGF. |
|  | Physical Modalities | Saini (2025) | Continuous therapeutic US (3 MHz, 2.0 W/cm², 5 minutes/session, 6 days/week for 2 weeks or until VAS ≤3) | Pulsed therapeutic US (3 MHz, 1.1 W/cm², 1:1 ratio, 5 minutes/session, 6 days/week for 2 weeks or until VAS ≤3) | Both continuous and pulsed US equally effective in reducing pain. |
|  | Needling | Zotelli (2017) | Sham acupuncture (nonpenetrating sham needle) | Real acupuncture (needle penetration) | Acupuncture was equally effective in reducing pain in both groups, increased unassisted painless mouth opening limitation only in the Treatment Group, and preserved Yin energy in the Treatment Group. |
|  | Physical Modalities | Madani (2019) | Sham laser (5 weeks, 2×/week, 10 sessions) | - LLLT (GaAlAs laser, 810 nm, 200 mW, 30 s/point, 21 J/cm², 5 weeks, 2×/week, 10 sessions)  - LAT (GaAlAs laser, 810 nm, 200 mW, 30 s/point, 21 J/cm², on ST6, ST7, LI4, 5 weeks, 2×/week, 10 sessions) | Both LLLT and LAT are effective in reducing pain and improving excursive and protrusive mandibular movements in TMD patients, with LAT offering a suitable alternative due to shorter chair time, though LLLT showed somewhat better results for TMJ pain and masseter insertion pain. |
|  | PT & EX, “Patient Education, Behavioral Therapy & Self-Care” | Nagata (2019) | Conventional treatment (self-exercise: molar pulldown type + S-MFT, CBT, education for TMD) | Conventional treatment + Jog-manipulation (4 types of manipulations with gauze pivot on last molar, repeated 3× if opening <40 mm, at first visit and subsequent visits until opening <40 mm) | The addition of jog-type mandibular manipulation to comprehensive conventional therapy, including improved therapeutic exercises and CBT, offers a short-term advantage in improving mouth-opening limitation, particularly after the initial treatment, but does not provide long-term superiority over conventional therapy alone for overall TMD symptoms, suggesting that well-executed self-exercises can achieve similar effects over time. |
|  | Injections | Lennartsson (2025) | Saline (2 injections, 3 months apart) | BoNT/A (100U, 2 injections, 3 months apart) | BoNT/A did not offer additional pain relief compared to saline for chronic orofacial myalgia in patients already receiving conservative treatment. |
|  | Oral Splints | Pho Duc (2016) | Conventional stabilization splint | CAD/CAM stabilization splint | Both CAD/CAM and conventional stabilization splints are equally efficacious in reducing various TMD symptoms, including pain, tension, and jaw joint noise, over a 9-month period, but neither significantly improves mandibular movements or condylar asymmetry, suggesting that the advanced manufacturing of CAD/CAM splints does not translate to superior clinical outcomes compared to conventional splints, although it may alleviate a wider range of symptoms. |
|  | “Patient Education, Behavioral Therapy & Self-Care” | Hwangbo (2023) | Conventional treatment (behavioral therapy, drug treatment, physical therapy) | Conventional treatment (behavioral therapy, drug treatment, physical therapy) + Smartphone application for CBT (5–10 min/day, daily use, for 5.9 weeks) | Repetitive CBT via a smartphone application, combined with conventional treatment, improved TMD symptoms, reducing pain and increasing mouth opening, suggesting its potential as a digital therapeutic. |
|  | Physical Modalities | Ekici (2022) | Placebo HILT (deactivated HIRO 3 device, pulsed emission 1064 nm, 15 sessions over 3 weeks; same 3-phase protocol as HILT group) | HILT (HIRO 3 device, pulsed emission 1064 nm, 3 kW peak power, 10.5 W mean power, 15 sessions over 3 weeks, 15 min/session, total 1029.2 J/session, 3-phase protocol on TMJ area and TPs) | HILT is an effective non-invasive treatment for myogenic temporomandibular joint disorder, demonstrating significant improvements in pain reduction, jaw function, disability, and QoL compared to placebo, particularly in the short and long term. |
|  | Oral Splints | Alajbeg IZ (2020) | Placebo splint (0.5 mm thin transparent foil, night-time use, 6 months) | Stabilization splint (full-coverage hard acrylic upper appliance, 1.5 mm thick, night-time use, 6 months) | Stabilization splint reduced pain-related disability, depressive symptoms, and oxidative stress markers. |
|  | Oral Splints, “Patient Education, Behavioral Therapy & Self-Care” | Giannakopoulos (2016) | Counselling only (waiting list for Michigan-type hard splint after 2 weeks) | Group A: Prefabricated oral splints (Aqualizer) worn during sleep and for at least 6 h/day (2 weeks)  Group B: Individualized vacuum-formed co-polyester oral splints worn during sleep and for at least 6 h/day (2 weeks) | Counselling combined with a vacuum-formed splint appears to be a favorable immediate short-term option for painful TMD symptoms. |
|  | Physical Modalities | Pihut (2018) | Biostimulation laser (808 nm, 32 J, 12 sessions, every other day, 3 min 45 s/session) + muscle self-exercises | ARS (full lower arch, 20 h/day, 4 months) | The ARS is an efficient tool for significantly decreasing pain associated with DDWR, demonstrating superiority over laser biostimulation and self-exercises in pain reduction over 16 weeks. |
|  | Injections | Sitnikova (2024) | SS injections | BoNT/A injections (50 U, single dose, bilateral into temporal and masseter muscles) | A 50-unit injection of BoNT/A might help with muscle pain symptoms, but the specific effect of the drug on pain compared to the placebo is not obvious. |
|  | Physical Modalities | Ekici (2022) | Placebo HILT (deactivated HIRO 3 device, pulsed emission 1064 nm, 15 sessions over 3 weeks; same 3-phase protocol as HILT group) | HILT (HIRO 3 device, pulsed emission 1064 nm, 3 kW peak power, 10.5 W mean power, 15 sessions over 3 weeks, 15 min/session, total 1029.2 J/session, 3-phase protocol on TMJ area and TPs) | HILT is an effective non-invasive treatment for myogenic temporomandibular joint disorder , demonstrating significant improvements in pain reduction, jaw function, disability, and QoL compared to placebo, particularly in the short and long term. |
| Myofascial pain | “Patient Education, Behavioral Therapy & Self-Care”, PT & EX | Aguiar (2023) | Conventional care (craniocervical manual therapy and exercise program) | Education-enhanced conventional care (Pain Science Education + craniocervical manual therapy and exercise program). PSE involved two 40-minute one-on-one sessions in weeks 1 and 2 | Education-enhanced conventional care improved TMD-related disability post-treatment and showed longer-lasting benefits for both pain and disability compared to conventional care alone. |
|  | Physical Modalities | Al-Quisi (2023) | Placebo (red LED light device applied without being switched on) | Red LED light (660 nm, 1.6 Watt, 5 min/point); LLLT (810 nm, 2.5 Hz, 1 Watt, 30 s/point) | Both effectively relieved pain associated with myogenic TMD with no significant differences in outcomes, but LED light is preferred due to biosafety and lower cost. |
|  | Needling | Aroca (2022) | Sham (Auricular acupuncture at Helix 3, 8 sessions over 8 weeks) | Auricular acupuncture (TMJ point 43.3, disposable semipermanent needles, 8 sessions over 8 weeks) OR OS (8 h daily/overnight, 8 weeks) | Auricular acupuncture at a specific TMJ ear point was effective in improving TMD symptoms, with results comparable to OS treatment. |
|  | “Patient Education, Behavioral Therapy & Self-Care”, Oral Splints | Costa (2015) | Counseling only | Counseling + stabilization appliance (night use, 5 months) | Counseling for masticatory myofascial pain reduced headache, which typically presented as long-lasting, bilateral frontotemporal, and pressing/tightening pain, with no extra benefit from an occlusal appliance. |
|  | “Patient Education, Behavioral Therapy & Self-Care”, Oral Splints | Costa (2015) | Counselling only | Counselling + OS (full-coverage hard acrylic upper stabilization appliance, worn at night, 5 months) | Minimally invasive strategies improved psychological aspects in TMD patients, and OS use appeared to hasten these effects. |
|  | Physical Modalities | Darwin (2024) | Th US: Therapeutic US (1 MHz, 1.3 W/cm², 10 mins, twice a week for 2 weeks) | TENS: TENS (75 Hz, 120 µs, 15 mins, twice a week for 2 weeks)  LLLT: LLLT (940 nm, 2.5 W, 900 J per TMJ, 6 mins, twice a week for 2 weeks) | All three therapies improved TMD symptoms, but LLLT showed superior efficacy in pain relief, mouth opening, and functional outcome compared to therapeutic US and TENS therapy. |
|  | Physical Modalities | De Oliveira Chami (2020) | Placebo LLLT (deactivated GaAlAs laser, identical application time/sound; 2 sessions, 48-h interval) | LLLT (GaAlAs laser, 808 nm, 100 mW, 80 J/cm², 22 s/site; 2 sessions, 48-h interval) | Laser treatment was effective in improving maximum oral opening and OHRQoL, while both laser and placebo reduced spontaneous pain with no significant intergroup difference in overall TMD, myofascial pain, mouth opening limitation, or OHRQOL. |
|  | Oral Splints | Deregibus (2021) | Mandibular OS (night-time use, ≥8 h/night, 6 months) | Upper Michigan OS (night-time use, ≥8 h/night, 6 months) | OSs (upper Michigan or mandibular) did not significantly reduce myofascial pain over six months, though mandibular splints showed superior improvement in specific lateral mandibular range of motion. |
|  | Needling, Pharmacotherapy, Oral Splints, PT & EX | Dunning (2024) | InterOS Therapy + Diclofenac + TMJ Mobilization (Interocclusal appliance night use for 4 weeks + Diclofenac 3 × 50 mg/day for 4 weeks, with possible reduction to 2 × 50mg/day + Non-thrust joint mobilization 10 min/session; up to 8 sessions, 1–2×/week for 4 weeks) | DN + Upper Cervical Spinal Manipulation (DN: 7 points, 20 min per session, needles in situ for 15–30 min; Upper Cervical Spinal Manipulation: targeting C0–C1, C1–C2, or C2–C3; up to 8 sessions, 1–2×/week for 4 weeks) | DN combined with upper cervical spinal manipulation was significantly more effective in reducing jaw pain intensity and increasing pain-free mouth opening in TMD patients compared to inter OS therapy, diclofenac, and TMJ mobilization over a 3-month period. |
|  | Physical Modalities | Ferreira (2017) | Placebo TENS (placebo device, allowed current for 40 s, then gradually reduced to be imperceptible, same electrode placement and parameters as active TENS, total 50 min, alternating low and high frequency—VHF with 4 Hz for first 25 min and 100 Hz for last 25 min) | Active TENS (Neurodyn Sapphire Compact Line, two calibrated channels, circular adhesive electrodes 3 cm, pulse duration <100 us, high intensities (below muscle contraction), total 50 min, alternating low and high frequency—VHF with 4 Hz for first 25 min and 100 Hz for last 25 min) | Short-term TENS therapy is superior to placebo in reducing facial pain and deep pain sensitivity, while also improving masticatory muscle EMG activity, specifically reducing activity during rest and increasing it during voluntary contraction and chewing. |
|  | PT & EX, Injections | García (2023) | MT (MT): Neuromuscular technique (masseter, SCM) + Jones technique/Ischemic compression (lateral pterygoid); 3 sessions, separated by 4 days | DN: Deep puncturing of 1–6 active MTrPs (masseter, lateral pterygoid, SCM) with 0.26 mm × 40 mm needles, local spasm responses; 3 sessions, separated by 4 days | DM are equally effective in reducing pain, improving active maximal mouth opening, decreasing cervical disability, and increasing pressure-pain thresholds in masseter and LPMs in patients with myofascial TMDs. |
|  | PT & EX | Gębska (2023) | TE, MT—Post-Isometric Muscle Relaxation (PIR) and TE (MTPIR_TE) | MT—Massage and TE (MTM_TE) | Soft tissue manual therapy and therapeutic exercises effectively reduce pain, improve masseter muscle bioelectrical function, and increase temporomandibular joint (TMJ) mobility in female patients with myogenic TMDs. Specifically, massage combined with therapeutic exercises demonstrates superior analgesic and myorelaxant effects, and greater improvements in lateral mandibular movements, compared to post-isometric muscle relaxation or therapeutic exercises alone. |
|  | PT & EX, “Patient Education, Behavioral Therapy & Self-Care” | Gębska (2024) | Counseling + TE (standardized exercise regimen, 6×/day, 10 reps each, 12 weeks) | KT (5-cm-wide tapes, Y-shaped patch over masseter muscle and TMJ area, 10–15% tension) + Counseling + TE; 12 days (excluding weekends) | KT combined with counseling and therapeutic exercises is an effective complementary treatment for female TMD patients, significantly improving pain, maximal mouth opening, and reducing perceived stress compared to counseling and exercises alone. |
|  | Oral Splints | Giannakopoulos (2018) | Conventional Michigan splint (night use) | Device-supported sensorimotor training (RehaBite® device, 3×/day, 15 min/session, 3 months) | Device-supported sensorimotor training is as effective as conventional splint therapy in short-term pain reduction for myofascial TMD patients with functional pain, but splint therapy is perceived as easier to use and more effective by patients. |
|  | Oral Splints, “Patient Education, Behavioral Therapy & Self-Care” | Hasanoglu Erbasar (2017) | Guidance, assurance, counselling, and behavioural changes | NTI-tss device + guidance, assurance, counselling, and behavioural changes (worn overnight, 6 weeks) | both groups improved in pain and jaw function, integrating an NTI-tss device did not provide additional benefit for myofascial pain symptoms. |
|  | Physical Modalities | Herpich (2017) | Placebo phototherapy | Combined phototherapy (super-pulsed laser 905 nm, red LED 640 nm, infrared LED 875 nm) | A single session of combined phototherapy significantly reduced pain intensity in women with TMD, with moderate effect sizes across different doses, but had no effect on PPT, EMG, or MMO. |
|  | Physical Modalities | Herpich (2019) | Sham PBMT | Active intraoral PBMT (super-pulsed laser 905 nm, red LED 670 nm, infrared LED 875 nm), applied to pterygoid muscles bilaterally, 6 sessions (3×/week for 2 weeks), total energy delivered 39.27 J/point, energy density 99.67 J/cm². | Intraoral PBMT significantly diminishes pain and improves functioning in women with myogenous TMD, but does not affect mandibular range of motion. |
|  | Oral Splints, “Patient Education, Behavioral Therapy & Self-Care”, PT & EX | Huttunen (2018) | Counseling and masticatory muscle exercises | Stabilization splint + counseling + masticatory muscle exercises | Depressive and nonspecific symptoms may negatively affect treatment outcome. |
|  | Oral Splints, Pharmacotherapy | Kahraman (2025) | Diclofenac sodium (75 mg, 2×/day, 2 weeks) + OS (8 hours/night, 3 months) | Vitamin D supplementation (10,000 IU, 1×/day, 8 weeks) + OS (8 hours/night, 3 months) | Vitamin D may be a viable alternative to NSAIDs for treating myofascial pain. |
|  | Physical Modalities | Kopacz (2020) | Not applicable (comparative study between two interventions) | MLT: LED light therapy with electromagnetic field (Viofor JPS, M1 method, P3 program, intensity 6, 10 min, 15 treatments over 3 weeks)  Cy: Cryotherapy (CryoFlex, CO2 system, −70 °C, 2 min, 15 treatments over 3 weeks) | Both LED light therapy with electromagnetic field (MLT) and Cy significantly reduced pain in TMD patients, with MLT showing a greater reduction in perceived pain. |
|  | Physical Modalities | Magri (2017) | Placebo LLLT; Without treatment | LLLT (780 nm, masseter and temporal = 5 J/cm², TMJ area = 7.5 J/cm², 8 sessions, twice a week) | The analgesia from LLLT in women with myofascial pain is due to non-specific effects during treatment, but active LLLT is more effective in maintaining analgesia after treatment for certain subgroups, while others do not respond. |
|  | Physical Modalities | Magri (2019) | Placebo LLLT | Active LLLT (780 nm, masseter and temporal = 5 J/cm², TMJ area = 7.5 J/cm², continuous emission, 8 sessions over 4 weeks, twice a week, direct contact) | Active and placebo LLLT effectively reduce pain in the short-term (up to six months post-intervention) for myofascial pain. |
|  | Oral Splints, Physical Modalities | Maracci (2020) | LLLT placebo | - Michigan OS (upper arch, 3 mm thick, night use) + adjustments  - LLLT (808 nm, 100 mW, 80 J/cm², 22 s/application, 2 sessions with 48-h interval) | Michigan OS is effective in reducing myofascial pain and improving OHRQoL, while the rapid LLLT protocol only improves QoL without significantly reducing myofascial pain. |
|  | Physical Modalities | Monaco (2019) | No treatment | ELIBA device (lingual elevator by Balercia, used ≥16 h/day for 6 months, constructed under ULF-TENS) | The ELIBA device effectively reduces myofascial TMD pain and improves jaw kinematics and sEMG values over 6 months in female patients who previously did not respond to ULF-TENS, demonstrating its potential as a treatment option for chronic TMD patients with insufficient freeway space for conventional orthotics, by promoting muscle relaxation and increasing interocclusal distance without occlusal modification. |
|  | Physical Modalities | Monteiro (2020) | Placebo (sham laser without activation) | PBMT (635 nm diode laser, 8 J/cm², 4 sessions over 4 weeks) | Laser therapy significantly reduced pain and improved mouth opening without side effects. |
|  | Needling | Moraes (2022) | Sham needling | DN (3 sessions, 7–14 day interval between sessions) | DN combined with counseling improved pain intensity, tinnitus, tinnitus discomfort, and reduced total THI score in patients with myofascial TMD and tinnitus. |
|  | Physical Modalities | Nadershah (2019) | Sham laser | PBMT therapy (940 nm diode, 257 J/treatment, total 1285 J, every 48 h for 10 days) | PBMT therapy is an effective short-term treatment for myofascial TMD pain, showing significant pain reduction compared to sham treatment. |
|  | Injections | Nitecka-Buchta (2018) | 0.9% NaCl (saline) intramuscular injection | 2 ml of Collagen MD Muscle intramuscular injection (days 0 and 7); Group II: 2 mL of 2% Lidocaine without vasoconstrictor intramuscular injection (days 0 and 7) | Intramuscular collagen injection is more efficient in reducing myofascial pain within masseter muscles than lidocaine injection, but further long-term trials are needed due to the short observation time. |
|  | Needling | Özden (2018) | Superficial DN (SDN) (5 mm depth, 3 sessions, once/week) | Deep DN (DDN) (≥10 mm depth, 3 sessions, once/week) | Superficial DN is more effective in reducing masseter-related myofascial TMD pain than deep DN, as measured by VAS scores and pressure pain threshold, although neither technique significantly improves maximal mouth opening. |
|  | PT & EX | Packer (2015) | Sham manipulation (placebo) | Upper thoracic manipulation (single session, T1 vertebral area) | Upper thoracic manipulation does not significantly affect vertical mouth opening or most masticatory muscle electromyographic activity in women with TMD, with only isolated immediate increases in some muscle activity during isometric depression, suggesting no substantial clinical effect. |
|  | PT & EX, Pharmacotherapy, “Patient Education, Behavioral Therapy & Self-Care” | Patil DJ Dheer DS (2023) | Standard therapy (NSAIDs + muscle relaxant for 1 week, physiotherapy, soft diet) | Standard therapy + CBT (6 weekly sessions of psychoeducation, distraction, imagination, self-suggestion, relaxation, cognitive restructuring, hypnosis training, assertiveness, relapse prevention) | Adding CBT to standard treatment enhances efficacy in TMD patients by reducing stress and improving QoL. |
|  | Physical Modalities, PT & EX | Percin (2025) | MT and EX (twice a week for 3 months) | aVNS/taVNS (bilateral, 20 min, 10 Hz, 250 µs pulse width, supra-threshold current, biphasic mode) + manual therapy and exercise (twice a week for 3 months) | aVNS/taVNS shows promising results as an adjunctive therapy for patients with TMD or myofascial pain. |
|  | Pharmacotherapy, Needling | Perez (2015) | Methocarbamol/paracetamol (2 tablets, every 6 hours, 3 weeks) | Deep DN (DDN) (3 applications, once per week, 3 weeks) of LPM TPs (TPs) | Deep DN of LPM TPs is more effective and safer than methocarbamol/paracetamol for reducing chronic myofascial pain, improving mandibular mobility (MMO, laterality, protrusion), and enhancing TMJ functionality, with benefits lasting up to 8 weeks post-treatment and fewer side effects. |
|  | “Patient Education, Behavioral Therapy & Self-Care”, PT & EX, Physical Modalities | Polat (2021) | Exercise + Patient Education | LLLT + TE + Patient Education, MPR + TE + Patient Education | LLLT and MPR combined with exercise are more effective than exercise alone for improving pressure pain thresholds in various masticatory and neck muscles in TMD patients, while all three approaches equally reduce TMD symptoms, improve jaw and head movements, decrease craniomandibular dysfunction, and improve psychological status and neck disability. |
|  | PT & EX | Rodriguez-Blanco (2015) | Neuromuscular technique over masseter muscles + passive hamstring muscle stretching | Neuromuscular technique over masseter muscles + passive hamstring muscle stretching + suboccipital muscle inhibition technique | The addition of a suboccipital muscle inhibition technique to a multimodal protocol combining masseter neuromuscular techniques and hamstring stretching does not significantly improve mouth opening, orofacial mechanosensitivity, or suboccipital and lumbar mobility in TMD patients with restricted condylar and C1 mobility. However, the experimental group did show significant intragroup improvements in suboccipital flexion and lumbar mobility as measured by the SAR test, and a trend towards better outcomes across several measures, suggesting a subtle benefit that did not reach statistical significance in intergroup comparison, possibly due to the immediate assessment design. |
|  | Oral Splints, Injections | Saglam (2024) | Healthy volunteers (Control Group 4) | - OS (OS) (Group 1)  - OS + masseter muscle lidocaine injection (Group 2)  - Masseter muscle lidocaine injection (Group 3) | While all treatments for myofascial pain are effective in reducing pain, increasing mouth opening, and decreasing masseter muscle stiffness, lidocaine injection alone offers quicker pain reduction, whereas the combination of OS and lidocaine injection is superior for sustained pain relief and greater improvements in MMO. |
|  | Physical Modalities | Sajedi (2022) | Placebo cupping/Sham laser | LLL acupuncture (808 nm, 0.5 W, 30 J, 4 J/cm², 60 s) or Cupping (10 cc disposable cups, 5 min) | Both low-level laser acupuncture and cupping are equally effective in reducing myofascial pain and improving mouth opening, but cupping offers a faster reduction in TPs and pain, while patients report higher satisfaction with low-level laser acupuncture due to fewer complications. |
|  | Physical Modalities | Sancakli (2015) | Placebo laser | - LLLT (820 nm, 3 J/cm², 300 mW, 10 s/point, 12 sessions) at greatest pain points (LGI)  - LLLT (820 nm, 3 J/cm², 300 mW, 10 s/point, 12 sessions) at pre-established points (LGII) | LLLT is effective in reducing masticatory muscle pain and improving mandibular function in TMD patients, with direct irradiation at the point of greatest pain showing numerically superior results compared to application at pre-established points, although not statistically significant, while placebo offers only slight non-significant improvements. |
|  | Physical Modalities, Oral Splints | Siqueira (2025) | OS (OS): stabilization splint (night use, 12 weeks) | L: PBMT (infrared light 808 nm, 3 J/cm² per point, 5 weekly sessions for 4 weeks);  OSL: OS (night use, 12 weeks) + PBMT (infrared light 808 nm, 3 J/cm² per point, 5 weekly sessions for 4 weeks) | Conservative treatments, especially the combination of PBMT therapy and OSs, effectively improved pain, mandibular mobility, and OHRQoL in individuals with muscular TMD. |
|  | Needling | Sousa (2025) | No control group (comparative study) | DN, Ischemic Compression, Cross-Taping | DN and ischemic compression are more effective for immediate relief of orofacial myofascial pain, with DN showing consistent long-term pain reduction and ischemic compression maintaining a slight reduction, while cross-taping shows an initial decrease in pain but an increase after one month, indicating it is less effective overall compared to the other two techniques. |
|  | PT & EX, “Patient Education, Behavioral Therapy & Self-Care”, Oral Splints | van Grootel (2017) | OS therapy (Michigan type, 12–30 weeks duration, 3–6 visits) | Physiotherapy (massage, cognitive-behavioral therapy, 10–21 weeks duration, 10–16 visits) | Physiotherapy may be preferred as initial therapy over OS therapy for myogenous TMD due to similar success rates and effectiveness but shorter duration. |
|  | Oral Splints, PT & EX | Lindfors (2020) | -No treatment (waiting list, 3 months)  -Stabilization appliance (hard acrylic, night use, 3 months) | - JEs (relaxation, free movements, resisted movements, stretching; 3×/day) | JEs effectively reduce pain, headache, and analgesic consumption in masticatory myofascial pain patients and are more cost-effective than stabilization appliances, despite similar pain reduction efficacy between the two active treatments. |
|  | PT & EX | Souza (2024) | Placebo treatment (turned-off therapeutic US) | Neck motor control training (8-week program, 30 min/week, + HEs 15–20 min/day) or MT (myofascial release, cervical mobilization, stretching, 30–40 min/week, + home self-massage/stretching) | Neck motor control training is highly effective for improving orofacial pain, jaw function, and OHRQoL in women with TMD, significantly outperforming placebo in all these aspects and manual therapy in QoL, although neither active treatment consistently improves jaw range of motion. |
| Headache attributed to TMD | “Patient Education, Behavioral Therapy & Self-Care”, Physical Modalities | Jo (2021) | Sham pulsed radiofrequency + conventional noninvasive TMD treatment (counselling for behavioral therapy, occlusal stabilization splint, thermal moist heat, US, low-level laser, transcutaneous electrical nerve stimulation, aceclofenac 100 mg bid for 14 days, cyclobenzaprine 10 mg qhs for 14 days) | Pulsed radiofrequency (1.5 W, 1 MHz, 10 min/session, once a week for 12 weeks, on both masticatory muscles) + conventional noninvasive TMD treatment (counselling for behavioral therapy, occlusal stabilization splint, thermal moist heat, US, low-level laser, transcutaneous electrical nerve stimulation, aceclofenac 100 mg bid for 14 days, cyclobenzaprine 10 mg qhs for 14 days) | Long-term regular pulsed radiofrequency therapy, combined with conventional treatments, effectively and durably reduced TMD pain and improved patient satisfaction with minimal discomfort. |
|  | Injections | Kim (2023) | Saline placebo injections | BoNT/A injections (Botulax®, Hugel Inc., 10 U/0.2 mL, total dose ≤150U, single injection) | Treatment with BoNT/A was relatively effective for masticatory muscle pain caused by TMDs and headache compared to the saline placebo. |
|  | Injections | Sitnikova (2024) | SS injections | BoNT/A injections (50 U, single dose, bilateral into temporal and masseter muscles) | A 50-unit injection of BoNT/A might help with muscle pain symptoms, but the specific effect of the drug on pain compared to the placebo is not obvious. |
|  | Injections | Lennartsson (2025) | Saline (2 injections, 3 months apart) | BoNT/A (100U, 2 injections, 3 months apart) | BoNT/A did not offer additional pain relief compared to saline for chronic orofacial myalgia in patients already receiving conservative treatment. |
| DDwR | Oral Splints, Physical Modalities | Abbasgholizadeh (2020) | Splint therapy | Group 2: Splint therapy + US-guided arthrocentesis (100–120 mL isotonic sodium chloride irrigation, 2 mL sodium hyaluronate injection, post-procedure splint use);  Group 3: Splint therapy + LLLT (Nd:YAG laser, 500 mW, 321 J/cm², 1064 nm, 1 min/point, 3×/week for 4 weeks) | All three treatments were effective, with splint therapy plus US-guided arthrocentesis providing faster initial improvement. |
|  | Injections | Haggag (2022) | Normal saline | 25% dextrose solution (intra-articular injection in two sites: superior joint space and retrodiscal tissue, 1mL per site, up to 4 injections at weekly intervals) | Intra-articular injection of 25% dextrose is effective, simple, easy, and safe for treating pain and dysfunction of TMJ DDWR, as evidenced by improved pain and MIO, and high patient satisfaction, and should be adopted when appropriate. |
|  | “Patient Education, Behavioral Therapy & Self-Care”, Physical Modalities, Oral Splints | Majid (2020) | Self-care instructions | - Physiotherapy (US therapy: 0.25 watts/cm², pulsed at 2–1, 2–3 mins/application, 3–4 times/week for 4 weeks) + self-care  - ARS (clear, hard acrylic, night-time for 3 months and 2 hours/day while awake for 3 months) + self-care | All three treatment modalities (self-care, physiotherapy + self-care, and ARS + self-care) were individually effective in alleviating DDwR symptoms in the short term, with no significant long-term differences in pain or muscle tenderness among them, though the ARS group demonstrated greater improvements in mouth opening and a unique reduction in TMJ clicking, suggesting that while monotherapy offers benefits, comprehensive care combining therapies may be a more practical initial choice for DDwR management considering cost-effectiveness and promptness. |
|  | Oral Splints, Injections, Physical Modalities | Rady (2022) | ARA | - Botulinum toxin type-A (single injection of 30 units)  - Low Level Laser Therapy (780 nm, 100 mW, 1.4 J/cm², 1.5 min, 3 TPs, 3 times/week for 3 months) | Both Botulinum Toxin Type-A and LLLT are effective alternatives to ARAs for treating symptomatic temporomandibular joint DDwR, as they significantly reduce joint pain and clicking while improving disc position and joint space index with faster symptom relief compared to the ARA. |
|  | Injections | Rezazadeh (2022) | Placebo injection (normal saline) | BoNT/A injection (15 U) into the LPM | BoNT/A injection reduced click and pain than control groups. |
|  | “Patient Education, Behavioral Therapy & Self-Care”, PT & EX | Simões (2023) | Counselling program (diet changes, avoid masticatory muscle overload, hot/cold compress, keep teeth separated, good posture, improve sleep, avoid parafunctional habits, decrease caffeine intake) | Counselling program + JEs (slow mouth opening/closing with tongue on incisors, pronouncing “N” with tongue on incisors, slow mouth opening/closing in mirror keeping midline straight, resisted jaw depression/elevation with hand) | Counselling plus JEs were more effective for TMJ click resolution, self-perception of treatment effectiveness, and palpation point improvement. |
|  | Pharmacotherapy | Tchivileva (2020) | Placebo | Propranolol (60 mg BID, 8-week maintenance after 1-week titration, 1-week taper) | Propranolol didn’t significantly change average facial pain, but it reduced pain by 30–50% and improved overall well-being in chronic TMD patients, proving safe and well-tolerated. |
|  | “Patient Education, Behavioral Therapy & Self-Care”, PT & EX | Wahlund (2021) | Information/counselling (PT/BT/SC) | JE + Information/counselling (PT/BT/SC) | The internet-delivered combined JE and information/counselling program is a more effective and credible treatment for adolescents with symptomatic temporomandibular joint DDwR than information/counselling alone, significantly improving painful catching/locking, eating ability, and jaw function limitation, along with increased jaw opening without clicking. |
|  | “Patient Education, Behavioral Therapy & Self-Care”, PT & EX, Oral Splints | Wänman (2019) | HE (jaw opening/closing in protruded position + isometric exercises) | - Bite splint (resilient, 4mm thick, night use)  - Supervised exercise (10 sessions: heat lamp, jaw opening-closing in protruded position, jaw opening/protrusion against resistance) | Both JE programs and bite splint therapy positively reduce perceived severity of TMJ clicking sounds, with supervised exercise and bite splint therapy showing greater reductions than HE, and supervised exercise additionally improving overall well-being by reducing TMD pain, neck disability, mood disturbances, and somatisation. |
| DDwoR | Arthrocentesis/Joint Lavage, PT & EX, Pharmacotherapy, “Patient Education, Behavioral Therapy & Self-Care” | Ritto (2022) | LA injection, soft diet, physiotherapy, CBT | Medication Group: LA injection, medication (tenoxicam) for 30 days, soft diet, physiotherapy, CBT  ALG: Arthrocentesis, soft diet, physiotherapy, CBT  ALMG: Arthrocentesis, medication (tenoxicam 20-mg pills) for 30 days, soft diet, physiotherapy, CBT | Both arthrocentesis and conservative modalities effectively reduced joint pain and increased mandibular opening. |
|  | Arthrocentesis/Joint Lavage, Oral Splints | Silva (2024) | Arthrocentesis (Group 2) | SIP (Group 1) | Both arthrocentesis and SIP effectively reduce pain and improve QoL parameters, but the splint is superior in restoring pain-free mouth opening, while arthrocentesis provides a quicker initial reduction in pain, suggesting that treatment choice should consider individual needs regarding speed of relief versus long-term functional opening. |
|  | Arthrocentesis/Joint Lavage | Folle (2018) | Conventional double puncture arthrocentesis (once, affected articulation; 300 mL saline lavage, followed by 1 mL sodium hyaluronate 10 mg infiltration in upper TMJ compartment; mandibular manipulation post-procedure) | Single puncture type 2 arthrocentesis (once, affected articulation, double-needle cannula; 300 mL saline lavage, followed by 1 mL sodium hyaluronate 10 mg infiltration in upper TMJ compartment; mandibular manipulation post-procedure) | Both conventional double puncture and single puncture type 2 arthrocentesis are equally effective in reducing pain and improving maximum interincisal distance in patients with temporomandibular joint DDwoR. |
|  | Arthrocentesis/Joint Lavage | Grossmann (2017) | Conventional two-needle arthrocentesis | Single-needle arthrocentesis with upper compartment distension | Both techniques were equally effective, but single-needle arthrocentesis is preferred due to its advantages for patients with painful hypomobilized TMJ. |
|  | Arthrocentesis/Joint Lavage | Grossmann (2021) | Two-needle arthrocentesis (classic positioning: 10 mm from tragus, 2 mm below corner-tragus line; 20 mm anterior to tragus, 10 mm inferior to corner-tragus line; associated with vacuum pump) | Two-needle arthrocentesis (parallel positioning: 10 mm from tragus, 2 mm below corner-tragus line; 14 mm anterior to tragus, 2 mm inferior to corner-tragus line; associated with vacuum pump) | Both TMJ arthrocentesis techniques reduced pain and improved mandibular movements, but the parallel needle positioning was faster and should be preferred. |
|  | Arthrocentesis/Joint Lavage, Oral Splints | Malekzadeh (2019) | Noninvasive (information, self-exercise, OSs) | Minimally invasive (information, arthrocentesis with lavage, manipulation, postoperative self-exercise) | Both noninvasive and minimally invasive interventions are equally effective for treating symptomatic closed lock. |
|  | “Patient Education, Behavioral Therapy & Self-Care”, Arthrocentesis/Joint Lavage | Malekzadeh (2019) | Noninvasive (information, self-exercise, OSs as needed) | Minimally invasive (information, arthrocentesis with lavage, manipulation, postoperative self-exercise) | Both noninvasive and minimally invasive treatments for symptomatic closed lock are equally effective, leading to similar improvements in mouth opening and pain reduction after one year; furthermore, patients with higher baseline maximal mouth opening and males are more likely to experience spontaneous recovery, suggesting a self-limiting course for some individuals. |
|  | Injections | Taleb (2025) | 0.9% normal saline injection | 5% Dextrose (D5W) injection | Single intra-articular 5% Dextrose prolotherapy is effective in rapidly and significantly relieving pain in female closed-lock patients, with a slower but significant improvement in mouth opening over 12 months, demonstrating superiority over normal saline injection for both pain and long-term mouth opening. |
|  | Arthrocentesis/Joint Lavage | Toameh (2019) | Arthrocentesis only (control group) | Arthrocentesis + HA, Arthrocentesis + PRP | Arthrocentesis combined with PRP is superior to HA or arthrocentesis alone in improving pain intensity and masticatory efficiency, while both PRP and HA significantly increase mouth opening more than arthrocentesis alone, suggesting PRP as the most effective overall treatment for DDwoR. |
|  | Injections | Pihut (2020) | Intra-articular injection of HA | Intra-articular injection of platelet rich plasma (0.4 mL for both joints, 3 times, 10-day intervals) | PRP and HA intra-articular injections are equally effective in significantly improving mouth opening and reducing pain intensity in patients with temporomandibular joint DDwoR, with both treatments showing substantial individual efficacy. |
|  | Physical Modalities | Yeladandi (2024) | TENS therapy | LLLT (980 nm, 1 Joule, 500 mW, 6 minutes, 8 sessions (2/week for 4 weeks)), US therapy (1.0 W/cm², pulsed mode, 1 MHz, 10 minutes, 12 sessions (1/day for 2 weeks)) | Both LLLT and US therapy are more effective than TENS in reducing TMD pain post-treatment, with LLLT showing superiority in pain reduction at mid-treatment; all three therapies are equally effective in improving mouth opening post-treatment, while LLLT and US show better mid-treatment mouth opening than TENS. |
|  | “Patient Education, Behavioral Therapy & Self-Care”, Oral Splints | Lei (2019) | Conservative treatment (patient self-care, moist heat, NSAIDs for pain, arthrocentesis for acute DDw/oR) | ARS therapy (upper arch splint, mandible in protrusive position, 24 h daily for 3 months, then night use) + Conservative treatment | ARS therapy is effective in promoting condylar repair and regeneration in adolescents/young adults with early-stage TMJ DJD, especially when an ideal disc-condyle relationship is established. |
|  | Arthrocentesis/Joint Lavage | Bhargava (2019) | Single-puncture arthrocentesis with modified double-lumen single-barrel needle (conventional) | US-guided single-puncture arthrocentesis using modified double-lumen single-barrel needle | US-guided single-puncture arthrocentesis is a promising method for TMJ lavage, requiring fewer needle manipulation attempts and less procedural time with comparable pain outcomes to the conventional method. |
|  | PT & EX, “Patient Education, Behavioral Therapy & Self-Care” | Nagata (2019) | Conventional treatment (self-exercise: molar pulldown type + S-MFT, CBT, education for TMD) | Conventional treatment + Jog-manipulation (4 types of manipulations with gauze pivot on last molar, repeated 3× if opening <40 mm, at first visit and subsequent visits until opening <40 mm) | The addition of jog-type mandibular manipulation to comprehensive conventional therapy, including improved therapeutic exercises and CBT, offers a short-term advantage in improving mouth-opening limitation, particularly after the initial treatment, but does not provide long-term superiority over conventional therapy alone for overall TMD symptoms, suggesting that well-executed self-exercises can achieve similar effects over time. |
|  | Injections | Lennartsson (2025) | Saline (2 injections, 3 months apart) | BoNT/A (100 U, 2 injections, 3 months apart) | BoNT/A did not offer additional pain relief compared to saline for chronic orofacial myalgia in patients already receiving conservative treatment. |
|  | Oral Splints | Pho Duc (2016) | Conventional stabilization splint | CAD/CAM stabilization splint | Both CAD/CAM and conventional stabilization splints are equally efficacious in reducing various TMD symptoms, including pain, tension, and jaw joint noise, over a 9-month period, but neither significantly improves mandibular movements or condylar asymmetry, suggesting that the advanced manufacturing of CAD/CAM splints does not translate to superior clinical outcomes compared to conventional splints, although it may alleviate a wider range of symptoms. |
| DJD | Arthrocentesis/Joint Lavage, PT & EX, Pharmacotherapy, “Patient Education, Behavioral Therapy & Self-Care” | Ritto (2022) | LA injection, soft diet, physiotherapy, CBT | Medication Group: LA injection, medication (tenoxicam) for 30 days, soft diet, physiotherapy, CBT  ALG: Arthrocentesis, soft diet, physiotherapy, CBT  ALMG: Arthrocentesis, medication (tenoxicam 20-mg pills.) for 30 days, soft diet, physiotherapy, CBT | Both arthrocentesis and conservative modalities effectively reduced joint pain and increased mandibular opening. |
|  | “Patient Education, Behavioral Therapy & Self-Care”, PT & EX | Giro (2016) | Kinesiographic assessment at T0, T1 (30 days) and T2 (60 days), with education and self-care instructions provided at T1 and reviewed at T2 | EG: Education instructions at T0, followed by education and self-care instructions at T1, and review at T2.  ESG: Education and self-care instructions at T0, with review at T1 and T2. Education included information about TMD, structures involved, causes, prognosis, and self-care for mandibular muscles. Self-care therapies included thermotherapy (moist heat pads, 15 min, 3×/day), mandibular exercises (controlled movements, stretching, resistance, coordination, 6 repetitions, 3×/day), and self-massage (masseter and temporal muscles, 3×/day) | ESG group positively influenced the mandibular movement pattern of women with chronic painful TMDs in the short-term. |
|  | “Patient Education, Behavioral Therapy & Self-Care”, Physical Modalities | Jo (2021) | Sham pulsed radiofrequency + conventional noninvasive TMD treatment (counselling for behavioral therapy, occlusal stabilization splint, thermal moist heat, US, low-level laser, transcutaneous electrical nerve stimulation, aceclofenac 100 mg bid for 14 days, cyclobenzaprine 10 mg qhs for 14 days) | Pulsed radiofrequency (1.5 W, 1 MHz, 10 min/session, once a week for 12 weeks, on both masticatory muscles) + conventional noninvasive TMD treatment (counselling for behavioral therapy, occlusal stabilization splint, thermal moist heat, US, low-level laser, transcutaneous electrical nerve stimulation, aceclofenac 100 mg bid for 14 days, cyclobenzaprine 10 mg qhs for 14 days) | Long-term regular pulsed radiofrequency therapy, combined with conventional treatments, effectively and durably reduced TMD pain and improved patient satisfaction with minimal discomfort. |
|  | Arthrocentesis/Joint Lavage, Pharmacotherapy | Yang (2018) | arthrocentesis 4 hyaluronate sodium injections + oral placebo for 3 months | arthrocentesis with 4 hyaluronate sodium injections + oral glucosamine hydrochloride (1.44 g/day) for 3 months | Oral glucosamine hydrochloride combined with hyaluronate sodium injection effectively relieved TMJ OA pain and improved TMJ functions. |
|  | Physical Modalities | Yeladandi (2024) | TENS therapy | LLLT (980 nm, 1 Joule, 500 mW, 6 minutes, 8 sessions (2/week for 4 weeks)), US therapy (1.0 W/cm², pulsed mode, 1 MHz, 10 minutes, 12 sessions (1/day for 2 weeks)) | Both LLLT and US therapy are more effective than TENS in reducing TMD pain post-treatment, with LLLT showing superiority in pain reduction at mid-treatment; all three therapies are equally effective in improving mouth opening post-treatment, while LLLT and US show better mid-treatment mouth opening than TENS. |
|  | “Patient Education, Behavioral Therapy & Self-Care”, Oral Splints | Lei (2019) | Conservative treatment (patient self-care, moist heat, NSAIDs for pain, arthrocentesis for acute DDw/oR) | ARS therapy (upper arch splint, mandible in protrusive position, 24 h daily for 3 months, then night use) + Conservative treatment | ARS therapy is effective in promoting condylar repair and regeneration in adolescents/young adults with early-stage TMJ DJD, especially when an ideal disc-condyle relationship is established. |
|  | Injections | Lennartsson (2025) | Saline (2 injections, 3 months apart) | BoNT/A (100 U, 2 injections, 3 months apart) | BoNT/A did not offer additional pain relief compared to saline for chronic orofacial myalgia in patients already receiving conservative treatment. |
| TMD | Oral Splints | Bergmann (2020) | AOS 3 month | Full-occlusion BFB splint (bruXane splint, vibratory stimulus with additional auditory alarm, 3 months | The tested BFB splint effectively reduced SB activity, particularly burst duration, and improved global pain perception and facial muscle pain, indicating it is a safe and effective treatment option for bruxism-related symptoms. |
|  | Oral Splints, “Patient Education, Behavioral Therapy & Self-Care” | Bijelic (2025) | Occlusal appliance therapy (OAT) | Internet-based behavioural therapy (IBT) (7-week online program with weekly phone feedback) | The current version of internet-based behavioral therapy (IBT) for adolescents with TMD pain was overly comprehensive and needs revision, as occlusal appliance therapy (OAT) showed better outcomes for pain intensity and pain-related disability. |
|  | Oral Splints, Physical Modalities | Celakil (2017) | OS (night use, 4 weeks) | Bio-oxidative ozone therapy (3 times/week, 10 min, 6 sessions over 2 weeks) | Both ozone therapy and OSs effectively reduced TMD pain and improved mandibular movements, but OSs showed superior objective pain relief based on pressure pain thresholds. |
|  | “Patient Education, Behavioral Therapy & Self-Care”, PT & EX | Delgado de la Serna (2019) | Physiotherapy alone (cranio-cervical and TMJ exercises, self-massage, patient education; 6 sessions over 1 month) | Physiotherapy + manual therapy (cranio-cervical and TMJ exercises, self-massage, patient education, plus cervico-mandibular manual therapies targeting TMJ and cervical/masticatory muscles; 6 sessions over 1 month) | Adding cervico-mandibular manual therapies to exercise and education significantly improved clinical, psychological, and physical outcomes for patients with somatic tinnitus attributed to TMD. |
|  | Physical Modalities | Furquim (2023) | Placeboo | PBMT therapy (PTB) with 780 nm wavelength | Applying PBMT therapy to pain points, identified by palpation, is more effective than application to pre-established points for reducing chronic pain in women with TMD, suggesting individualized protocols based on pain palpation. |
|  | Oral Splints | Gerstner (2020) | “Michigan” bite splint (nightly use, 4 months) | SOVA bite splint (over-the-counter, “boil-and-bite” type, self-fabricated to clinically acceptable standards, nightly use, 4 months) | OTC splints show potential for curbing SB impacts, but active dental oversight during self-fabrication is strongly recommended. |
|  | Physical Modalities | Godoy (2017) | Sham LLLT | Active LLLT (780 nm, 25 J/cm², 50 mW, 20-second exposure per point, 12 sessions, 2×/week, 3 points over masseter, 1 point over anterior temporal muscle per side) | Further studies with a larger sample size are needed to confirm the present findings regarding LLLT for TMD. |
|  | Physical Modalities, PT & EX | Javed (2024) | Bowen’s Therapy + US (Mode 1:1, 3 MHz, 1.5 W/cm², 5 min, 2 sessions/week) + Taping (y-shape). Bowen’s technique on TMJ, masticators, temporalis muscles, 2–3 repetitions in 1 set, 4–5 sets per session. Total duration 4 weeks | PIR + US (Mode 1:1, 3 MHz, 1.5 W/cm², 5 min, 2 sessions/week) + Taping (y-shape). EX (mouth open, close, right/left lateral deviations) 5 times per session, 5-second hold and rest, 2 times/week for 4 weeks. | PIR was more effective than Bowen’s therapy in improving pain, mouth opening range of motion, and functional activity. |
|  | Physical Modalities | Machado (2016) | Healthy subjects (n = 20) | - LLLT + OM-exercises (GI)  - Orofacial MFT (OMT) (GII)  - Placebo LLLT + OM-exercises (GIII)  - LLLT alone (GIV) (LLLT: AsGaAl; 780-nm; 60 mW; 40 s; 60 ± 1.0 J/cm²; 12 sessions over 120 days) | LLLT combined with oral motor exercises is more effective for TMD rehabilitation than LLLT alone, and treatments combining pain relief strategies with oral motor exercises are promising for reducing TMD symptoms and improving functional recovery. |
|  | Oral Splints | Nagata (2015) | Non-splint multimodal therapy (NS) | Non-splint multimodal therapy (NS) + Stabilisation splint (hard, clear, acrylic resin, upper teeth, worn every night while sleeping, 2 weeks to apply, 6–10 weeks of evaluation) | Stabilisation splint therapy combined with non-splint multimodal therapy is not superior to non-splint multimodal therapy alone for treating RDC/TMD Axis I patients in terms of mouth-opening limitation, oro-facial pain, and TMJ sounds, with a minor exception for pain reduction in DJD patients, thus not supporting the additional effects of stabilisation splints for TMD patients receiving multimodal therapy. |
|  | Physical Modalities | Nahar (2023) | TENS therapy | LLLT (diode laser system, 650–900 nm wavelength, 0.5 watts for 30 seconds, noncontact mode, scanning motion, 10 minutes, 2×/week for 2 weeks) | Both LLLT and TENS effectively reduce pain, increase mouth opening, and decrease muscle tenderness in TMD patients, with LLLT showing slightly superior efficacy, particularly in muscle tenderness remission. |
|  | PT & EX, Physical Modalities, Oral Splints, Occlusal Equilibration Therapy | Nemani (2024) | Cervical physiotherapy | - Soft splints + cervical physiotherapy + occlusal equilibration (Group 2),  - TENS + cervical physiotherapy + occlusal equilibration (Group 3),  - LLLT + cervical physiotherapy + occlusal equilibration (Group 4) | TENS and LLLT are equally effective in reducing TMJ pain, outperforming soft splints and cervical physiotherapy alone, but none of the interventions significantly reduced cervical pain or showed a correlation between TMJ and cervical pain. |
|  | Physical Modalities, PT & EX | Patil and Aileni (2017) | Not applicable (comparative study between two active treatments) | TENS group: TENS (HiDow FDA CLASS II Approved Wireless TENS/EMS system, 20 W, max frequency 60 Hz, amplitude 1–10 µA), 30 minutes, once a week for 4 weeks  HE group: HE program (active/passive jaw opening/closing, isometric, stretching, resistive JEs), 6 seconds per exercise, 10 repetitions, twice a day for 4 weeks | Both TENS and HE therapies were effective in reducing masticatory muscle and joint pain and improving mouth opening for TMD patients, with TENS showing better pain reduction. |
|  | “Patient Education, Behavioral Therapy & Self-Care”, Oral Splints, Needling, PT & EX | Peixoto (2021) | Counseling (C) | - OS (OS) (night use)  - SA (8 sessions, 40 min each, 2×/week for 4 weeks)  - MT (MT) (thermal agents + therapeutic exercises, 40 min/session, 2×/week for 4 weeks) | SA, OSs, and manual therapy are effective in reducing short-term pain in TMD patients, with manual therapy showing superiority over counseling for pain reduction; however, SA is not effective in improving sleep or QoL, unlike OSs and manual therapy, which improve sleep and specific QoL domains, respectively, indicating SA as a viable pain relief alternative but not a comprehensive solution for TMD-related sleep and QoL issues. |
|  | “Patient Education, Behavioral Therapy & Self-Care”, Oral Splints | Ram (2021) | Education for SM and counseling | - Muscle energy technique  - OS therapy  - Muscle energy technique + OS therapy (combined treatment) | Muscle energy technique, OS therapy, and combined treatment are all effective in reducing TMD pain compared to SM and counseling alone, with muscle energy technique and combined treatment showing superior improvements in mouth opening over both OS therapy and SM and counseling, suggesting the added benefit of active exercises. |
|  | “Patient Education, Behavioral Therapy & Self-Care”, Oral Splints, PT & EX | Resende (2019) | OS (OS) OR MT (MT) OR Counseling (C) | OS + Counseling (OSC; combination of both previous protocols) | In the short-term (1 month), conservative therapies for TMD (OSs, manual therapy, counseling, or their combination) effectively improved pain, sleep quality, and QoL, but no single therapy proved superior to the others. |
|  | Physical Modalities, PT & EX | Rezaie (2022) | Routine conservative treatment (TENS, US, gentle massage for 25 mins over 10 sessions) | Routine conservative treatment + MT of TMJ and upper cervical spine (soft tissue release, mobilization) for 10 sessions | Adding manual therapy of the upper cervical spine and TMJ to routine conservative treatment effectively relieved pain and increased MMO and cervical flexion in patients with TMJDs. |
|  | Physical Modalities, Oral Splints | Rodríguez (2024) | OS (rigid removable appliance, 24 h/day use for 3–4 months) | Group A: Transcutaneous electrostimulation (KWD-808 equipment, 210 Hz low frequency, 10–140 Hz high frequency, 30–50 volts amplitude, 40–100 msec pulse duration, 0.70 output intensity for 100 Hz, 20 min per treatment) + OS (24 h/day use)  Group B: Percutaneous electrostimulation (KWD-808 equipment, disposable sterile steel acupuncture needles, 210 Hz low frequency, 10–140 Hz high frequency, 30–50 volts amplitude, 40–100 msec pulse duration, 0.70 output intensity for 100 Hz, 20 min per treatment) + OS (24 h/day use) | Transcutaneous electrostimulation as an adjuvant to OSs is a feasible and faster alternative for treating TMDs, significantly reducing muscle fatigue, joint pain, and muscle pain. |
|  | Occlusal Equilibration Therapy | Santana-Penín (2023) | Sham therapy (non-cutting rotary instrument, 2 sessions: 90 min & 30 min) | Occlusal Equilibration Therapy (minimal invasive occlusal remodeling, 2 sessions: 90 min & 30 min) | Equilibration therapy significantly reduced facial pain associated with chronic TMDs and increased maximum unassisted mouth opening compared to sham therapy over six months, with no serious adverse events. |
|  | Injections | Şen (2020) | Acupuncture on non-specific points | Acupuncture on specific points (5 local: BL-2, BL-3, SI-19, ST-7, TE-21; 2 distal: BL-34, SI-3; + ear acupuncture; 14 needles, 1 cm depth, 45 min, 1×/week for 4 weeks; patient rotation every 15 min + self-stimulate ear needles 3×/day) | Acupuncture, regardless of specific or non-specific point selection, effectively reduces non-chronic TMD pain and improves OHRQoL; however, specific acupuncture additionally reduces depressivity, while non-specific acupuncture offers a significant increase in mouth opening. Despite similar overall efficacy in pain reduction, the pattern of pain reduction differs between specific and non-specific points, with specific points demonstrating earlier pain relief. |
|  | Arthrocentesis/Joint Lavage | Singh (2017) | Arthrocentesis alone | Arthrocentesis + duloxetine (30 mg twice daily for 12-week therapy) | Arthrocentesis combined with duloxetine is more effective in reducing TMJ pain and improving jaw mobility than arthrocentesis alone, although duloxetine does not impact anxiety, depression, or IL-6 levels. |
|  | “Patient Education, Behavioral Therapy & Self-Care”, PT & EX, Oral Splints | Wahlund (2017) | Information only (control) | - OS  - Relaxation training | Treatment condition and gender are consistent predictors of clinically significant outcomes in adolescents with TMD, with occlusal appliances being more effective than relaxation training/control, and boys showing better responses than girls; additionally, shorter TMD pain history and lower analgesic consumption are important long-term predictors of favorable outcomes. |

ALG: Arthrocentesis plus local anesthetic group; ALMG: Arthrocentesis plus local anesthetic and medication group; OS: Occlusal Splint; aVNS: Auricular Vagus Nerve Stimulation; taVNS: Transcutaneous Auricular Vagus Nerve Stimulation; ARS: Anterior Repositioning Splint; BFB: Biofeedback; BST: Behavioral and Self-care Therapy; BoNT/A: Botulinum Toxin Type A; CAD/CAM: Computer-Aided Design/Computer-Aided Manufacturing; CBT: Cognitive Behavioral Therapy; CS: Corticosteroid; CT: Control Therapy; CY: Cryotherapy; DDwR: Disc Displacement with Reduction; DDwoR/DDw/oR: Disc Displacement without Reduction; DF: Diacutaneous Fibrolysis; DJD: Degenerative Joint Disease; DM: Dry Needling and Manual therapy; DN: Dry Needling; EGF: Epidermal Growth Factor; EG: Education Group; EMG: Electromyography; ESG: Education and Self-care Group; EX: Exercises; GCM: Glucosamine, Chondroitin Sulfate, and Methylsulfonylmethane; HA: Hyaluronic Acid; HE: Home Exercise; HILT: High-Intensity Laser Therapy; JE: Jaw Exercise; KT: Kinesio Taping; LAT: Laser Acupuncture Therapy; LA: Local Anesthetic; LLLT: Low-Level Laser Therapy; LPM: Lateral Pterygoid Muscle; MFT: Myofunctional Therapy; MIO: Maximal Interincisal Opening; MMO: Maximum Mouth Opening; MPA: Methylprednisolone Acetate; MPR: Manual Pressure Release; MRI: Magnetic Resonance Imaging; MT: Manual Therapy; MTPIR_TE: Manual Therapy with Post-Isometric Relaxation and Therapeutic Exercises; MVIC: Maximal Voluntary Isometric Contraction; NSAIDs: Nonsteroidal Anti-inflammatory Drugs; NTI-tss: Nociceptive Trigeminal Inhibition Tension Suppression System; OS: Occlusal Splint; OHRQoL: Oral Health-Related Quality of Life; PBMT: Photobiomodulation Therapy; PET: Polyethylene Terephthalate; PIR: Post-Isometric Relaxation; PLLT: Piano Level Laser Therapy; PRP: Platelet-Rich Plasma; PSWT: Pulsed Short Wave Therapy; PT: Physical Therapy; rTMS: Repetitive Transcranial Magnetic Stimulation; SA: Scalp Acupuncture; SB: Sleep Bruxism; SIP: Stabilizing Interocclusal Splint; SLE: Systemic Lupus Erythematosus; SM: Self-Management; S-MFT: Simplified Myofunctional Therapy; SS: Saline Solution; TA: Therapeutic Approach; TENS: Transcutaneous Electrical Nerve Stimulation; TE: Therapeutic Exercises; TMD: Temporomandibular Disorder; TMJ: Temporomandibular Joint; TP: Trigger Point; US: Ultrasound; VAS: Visual Analog Scale; OA: osteoarthritis; qhs: every night at bedtime; JEs: jaw exercises; RMT: relaxation muscle training; EVA: visual analog scale (Spanish/French abbreviation commonly used for VAS); HIRO: high-intensity red/infrared irradiation; TPs: trigger points; LED: light-emitting diode; MTrPs: myofascial trigger points; LPMs: lateral pterygoid muscles; ULF: ultra-low frequency; NaCl: sodium chloride; SAR: suboccipital flexion–extension angle ratio; LGI: laser group I; ARA: active range of motion; BID: twice daily; SC: self-care; IL-6: interleukin-6.
